# Compressed Cells Facilitate Adhesion Through Glycocalyx

**DOI:** 10.1002/advs.202413586

**Published:** 2025-08-15

**Authors:** Xiaole Wang, Jonne Helenius, Daniel J. Müller, Nico Strohmeyer

**Affiliations:** ^1^ Department of Biosystems Science and Engineering Eidgenössische Technische Hochschule (ETH) Zurich Klingelbergstrasse 48 Basel 4056 Switzerland

**Keywords:** cell adhesion initiation, collagen, compression, fibronectin, glycocalyx, integrin, single‐cell force spectroscopy

## Abstract

Exposed to mechanical confinement, mammalian cells can establish remarkable unspecific adhesion, which is independent of integrins. How cells facilitate such adhesion remains unclear. Here, it is investigated how mammalian cells exposed to compression initiate unspecific and integrin‐mediated adhesion. It is observed that with increasing compression, cells increase adhesion to collagen I or fibronectin and strengthen adhesion faster. Under low and medium compression, cells minimally increase unspecific adhesion to substrates that lack specific binding sites for cell surface receptors, such as integrins. However, under high compression, mammalian cells switch to a strong unspecific adhesion state, which significantly contributes to cell‐extracellular matrix (ECM) adhesion. Thereby cells use the glycocalyx to directly facilitate strong unspecific adhesion and to enhance early integrin‐mediated adhesion. The mechanistic insight of how cells unspecifically adhere to substrates under confinement opens avenues to better understand cell adhesion in development, homeostasis, disease, and in a wide range of biotechnological and medical applications in which cells are exposed to mechanical confinement.

## Graphical abstract

This study quantifies how cell compression and confinement triggers cell adhesion. Under low compression, integrins dominate cell adhesion to extracellular matrix (ECM) proteins, whereas the glycocalyx contributes weak unspecific adhesion. With increasing compression, the glycocalyx increases its unspecific adhesion to ECM and non‐ECM proteins and can contribute considerably to the integrin‐mediated cell adhesion to the ECM.

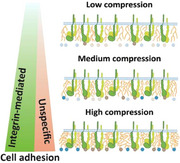

## 1 Introduction

Mammalian cells in tissue are constantly exposed to mechanical confinement and compression, under which they show remarkable ability to establish integrin‐mediated specific and integrin‐independent adhesion in order to migrate in the extracellular matrix (ECM).^[^ ^1^, ^2^, ^3^, ^4^, ^5^ ^]^ The ECM is a complex and dense network of mainly fibrous proteins, such as collagens, fibronectin, laminins, and proteoglycans.^[^ ^6^, ^7^, ^8^ ^]^ Serving as scaffold for cells within tissues, the ECM provides essential structural, mechanical, and biochemical information that regulates cell state and function.^[^ ^7^, ^9^ ^]^ Although the underlying principles of force generation in both integrin‐mediated and ‐independent migration are similar, relying on the dynamic and contractile actomyosin cortex,^[^ ^10^, ^11^, ^12^ ^]^ their friction‐generating adhesion mechanisms to the ECM are fundamentally different. For integrin‐mediated migration, mesenchymal cells require a subset of 24 different α/β heterodimeric integrins to physically anchor the actin cytoskeleton to the ECM.^[^ ^13^, ^14^ ^]^ Integrins assemble large supramolecular adhesion sites, collectively called adhesomes,^[^ ^15^, ^16^ ^]^ that transduce force to integrins by actin retrograde flow or actomyosin contractility,^[^ ^17^, ^18^, ^19^, ^20^ ^]^ and recruit actin polymerization machineries and activate actin regulators to trigger cell spreading and migration.^[^ ^21^, ^22^ ^]^ Integrin‐mediated cell‐ECM adhesion is essential for the migration of several cell types in vivo and for unconfined migration in vitro.^[^ ^23^, ^24^, ^25^, ^26^ ^]^ However, in confined spaces, cells can employ a fast ameboid migration mode that is driven by the contractile actomyosin cortex, minimal adhesion to the environment, and the ability to squeeze through small spaces using cell membrane blebs.^[^ ^10^, ^27^ ^]^ The forces driving the cell forward are significantly lower compared to those in integrin‐mediated migration, and the force distribution in integrin‐independent migration is reversed, causing the substrate to expand rather than contract in the direction of movement.^[^ ^1^ ^]^ Mesenchymal cells can switch from integrin‐mediated to integrin‐independent migration under highly confined conditions.^[^ ^28^, ^29^ ^]^ Interestingly, in vitro cells that are compressed between two surfaces to which they do not commonly adhere, such as polyethylene glycol (PEG), also show this amoeboid migration.^[^ ^2^, ^30^ ^]^ However, little is known about how cells establish integrin‐independent adhesion to their environment, which is essential for locomotion. Furthermore, it remains unclear how compression regulates integrin‐mediated and integrin‐independent adhesion.

The glycocalyx, which is a layer of sugars covering mammalian cells that predominantly originates from glycoproteins and glycolipids, emerges as regulator of cell‐ECM adhesion.^[^ ^31^ ^]^ Although the exact contributions of the glycocalyx in regulating cell‐ECM adhesion remain unclear, modulation of the biophysical properties of the glycocalyx changes the organization and function of integrins in adhesion sites.^[^ ^32^, ^33^ ^]^ Further, the glycocalyx implicates in mediating generic cell‐ECM adhesion in an integrin‐independent manner and cell–cell adhesion.^[^ ^34^, ^35^ ^]^ However, whether and how the glycocalyx synergizes with integrins to establish early cell adhesion and early mechanosensing remains largely unknown.

Here, we employ atomic force microscopy (AFM)‐based single‐cell force spectroscopy (SCFS) to quantify cell adhesion forces and to characterize how cell compression regulates integrin‐mediated and ‐independent adhesion initiation.^[^ ^36^, ^37^ ^]^ In addition, we characterize the participation of the glycocalyx in adhesion initiation. We find that cells increase adhesion force and accelerate adhesion strengthening with compression. Moreover, at high compression force, cells switch to a different adhesion state, to which the glycocalyx contributes considerably.

## 2 Results

### 2.1 Cells Initiating Adhesion Respond to Compressive Force

**Figure 1:**
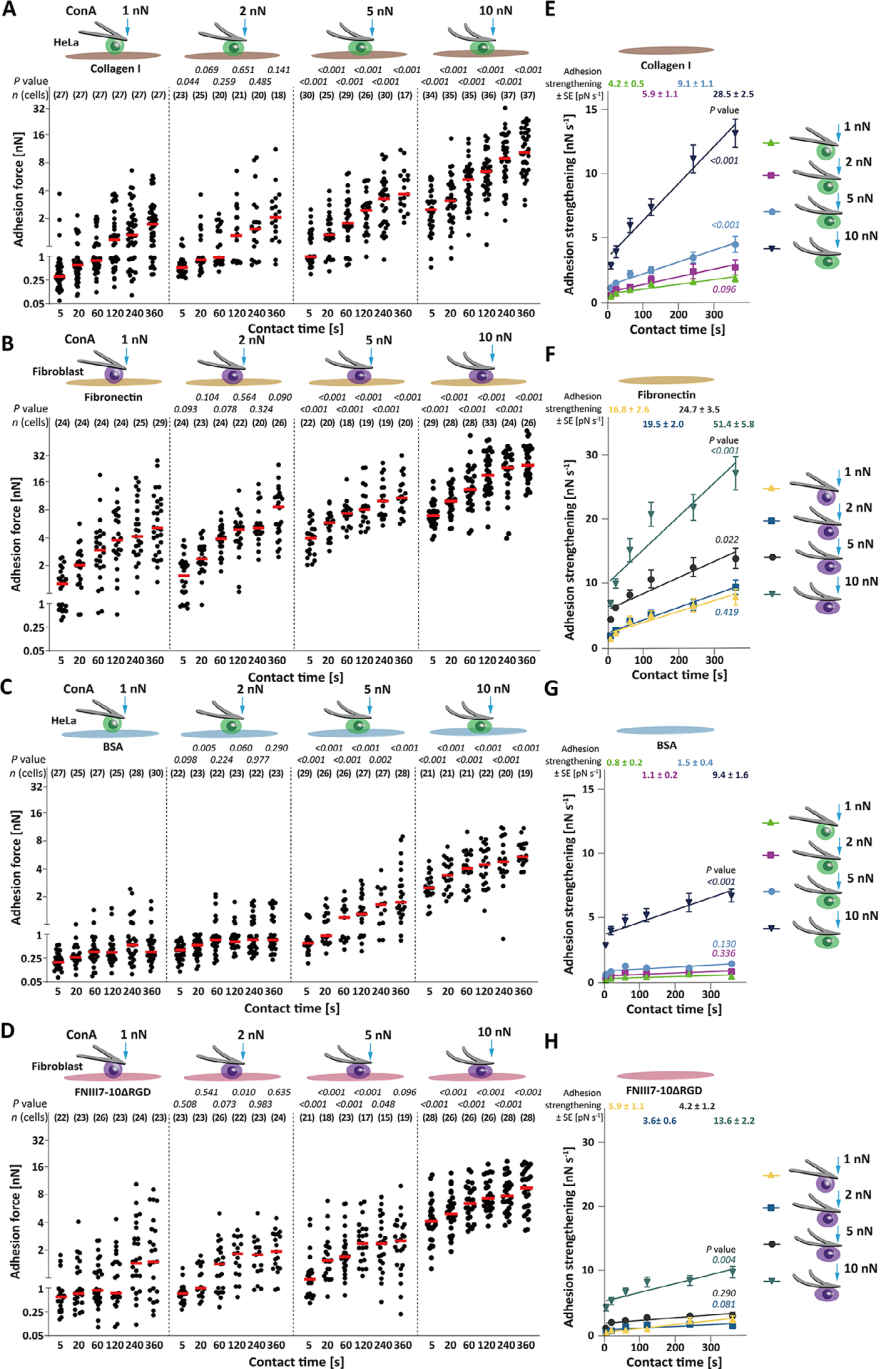
Adhesion forces of HeLa cells and fibroblasts correlate with compression force. A–D) Adhesion force of A) HeLa cells to collagen I, B) fibroblasts to fibronectin, C) HeLa cells to BSA, and D) fibroblasts to FNIII7‐10ΔRGD at compression forces of 1, 2, 5, and 10 nN. Dots represent adhesion forces of single cells, red bars median values, and *n*(cells) the number of independent cells tested in at least three independent experiments. *p* values were calculated using two sided Mann–Whitney tests and compare the adhesion force of cells under the given compression with the adhesion force of HeLa cells to A) collagen I or C) BSA and of fibroblasts to B) fibronectin or D) FNIII7‐10ΔRGD substrates at 1 nN compression. Statistical analysis comparing the potential difference between adhesion force of the displayed data under different compression forces is given in Tables S1 and S2 (Supporting Information). E–H) Adhesion strengthening rates of E) HeLa cells to collagen I, F) fibroblasts to fibronectin, G) HeLa cells to BSA, and H) fibroblasts to FNIII7‐10ΔRGD at compression forces of 1, 2, 5, and 10 nN. The rates were quantified as the slope of a linear regression fit of cell adhesion forces for all contact times (data taken from Figure 1A–D). Values give best fit values of adhesion strengthening rates ± standard errors (SE). Dots depict mean adhesion forces, error bars SEM, and lines linear regressions. *p* values were calculated by extra sum squares *F* test and compare the adhesion strengthening rate under the indicated compression force and 1 nN compression force. Statistical analysis adhesion strengthening rates of cells at different compression forces is given in Table S3 (Supporting Information).

To investigate how compression affects cells in initiating adhesion to substrates exposing and to substrates lacking integrin specific binding sites, we applied AFM‐based SCFS to quantify the adhesion force of a cell subjected to different compression forces (Figure S1, Supporting Information). Thereto, we attached a single rounded wild‐type (wt) HeLa cell or wt mouse embryonic fibroblast to a concanavalin A (ConA)‐coated AFM microcantilever. Next, we used the microcantilever to bring the rounded cell into contact with a substrate‐coated Petri glass dishes until reaching a preset compression force. Upon reaching this compression, the cantilever was kept at constant height and the cell was allowed to establish adhesion to the substrate for contact times ranging from 5 to 360 s. Subsequently, we measured the cell adhesion force by mechanically separating cell and substrate (**Figure** **1**). For integrin specific substrates we coated the glass with collagen I for HeLa cells and full‐length fibronectin for fibroblasts. As unspecific substrates, we used BSA for HeLa cells and the fibronectin fragment FNIII7‐10ΔRGD lacking an integrin binding site for fibroblasts. In SCFS, we applied low (1 and 2 nN), medium (5 nN), or high (10 nN) setpoint compression forces (Figure S2A, Supporting Information). Due to the viscoelastic response of the single fibroblast to the setpoint compression force,^[^ ^38^ ^]^ the compression force decreased during the contact time, until a plateau compression force was reached. For both cell lines, the compression force at the end of the contact time linearly scaled with the setpoint compression force for all contact times (Figure S2B,C, Supporting Information). Rounded HeLa cells were compressed by 1.6 ± 0.8 µm (mean ± SD, 9.9% of cell height) at 1 nN, 2.0 ± 0.8 µm (13.0% of cell height) at 2 nN, 3.2 ± 0.7 µm (20.0% of cell height) at 5 nN, or 4.2 ± 0.8 µm (26.2% of cell height) at 10 nN (Figure S3A–C, Supporting Information). Rounded fibroblasts were compressed by 1.3 ± 0.8 µm (8.6% of cell height) at 1 nN, 1.6 ± 0.9 µm (10.7% of cell height) at 2 nN, 2.0 ± 1.2 µm (12.9% of cell height) at 5 nN, or 2.7 ± 1.6 µm (17.4% of cell height) at 10 nN. We optically verified minimal changes of the cell height and the cell‐substrate contact area during 360 s contact time (Figure S3D–F, Supporting Information). Increasing the compression force increased the externally applied pressure on HeLa cells to 8.3 ± 3.1 Pa (1 nN), 15.2 ± 4.7 Pa (2 nN), 32.3 ± 12.7 Pa (5 nN), and 58.3 ± 18.9 Pa (10 nN) and on fibroblasts to 10.7 ± 3.2 Pa (1 nN), 20.7 ± 4.6 Pa (2 nN), 41.6 ± 8.2 Pa (5 nN), and 71.6 ± 8.9 Pa (10 nN; Figure S3G,H, Supporting Information).

With increasing compression and contact time, HeLa cells and fibroblasts increased the adhesion force to collagen I and fibronectin (Figure 1A,B). At low compression, the adhesion forces of both cell lines were compression force independent. However, they considerably increased the adhesion force at medium and high compression (Tables S1 and S2, Supporting Information). Surprisingly, despite the relatively small increase in contact area from ≈184 µm^2^ at medium to ≈208 µm^2^ (≈1.1‐fold) at high compression, HeLa cells increased the adhesion force to collagen I ≈3‐fold. Similarly, when increasing from medium to high compression, fibroblasts increased their contact area with fibronectin from ≈119 to ≈132 µm^2^ (≈1.1‐fold) and their adhesion force ≈2‐fold. This considerable increase in adhesion force by two to threefold together with the minimal increase in contact area (≤1.1‐fold), suggests that compressed cells upregulate integrin‐mediated adhesion.

Next, we analyzed the adhesion force quantified of HeLa cells to BSA and of fibroblasts to FNIII7‐10ΔRGD at different compression forces (Figure 1C,D; Tables S1 and S2, Supporting Information). Since HeLa cells and fibroblasts cannot adhere to these substrates via integrins, the cell adhesion force is integrin‐independent and from here on called unspecific adhesion. Exposed to low compression, HeLa cells and fibroblasts established minimal unspecific adhesion force to BSA and FNIII7‐10ΔRGD, respectively. At medium compression, HeLa cells substantially increased the unspecific adhesion force to BSA, while fibroblasts slightly increased the unspecific adhesion force. Surprisingly, when increasing from medium to high compression, both cell lines increased the unspecific adhesion force ≈5‐fold, despite the minimal increase in contact area between cell and substrate (≤ 1.1‐fold). Regardless of compression, the cell adhesion force to ECM proteins was always higher than that to FNIII7‐10ΔRGD or BSA.

Together, the results indicate that cells under compression considerably increase their adhesion to ECM proteins. However, under high compression, HeLa cells and fibroblasts also considerably increase their unspecific adhesion to proteins to which they only weakly adhere under low and medium compression.

### 2.2 Compression Accelerates Cell Adhesion Strengthening

Next, we characterized how HeLa cells and fibroblasts under compression strengthen adhesion. Thereto, we quantified the increase of the cell adhesion force over contact time as the slope of a linear regression fit (Figure 1E–H). Under low compression, the adhesion strengthening of HeLa cells and fibroblasts to collagen I or fibronectin was compression force independent (Figure 1E,F; Table S3, Supporting Information). Under medium compression, the adhesion strengthening rate of HeLa cells to collagen I increased ≈1.4‐fold. Similarly, compared to low compression, the adhesion strengthening rate of fibroblasts to fibronectin increased ≈1.5‐fold at medium compression. Upon further increasing the compression from medium to high, the adhesion strengthening rate of HeLa cells to collagen I increased ≈3.1‐fold. Simultaneously, the adhesion strengthening rate of fibroblasts increased ≈2.1‐fold. The results indicate that the compression of cells not only increases their adhesion force to ECM proteins but also accelerates their adhesion strengthening.

At low and medium compression, both cell lines strengthened the unspecific adhesion to BSA or FNIII7‐10ΔRGD at minimal rates, while their adhesion strengthening was compression force independent (Figure 1G,H; Table S3, Supporting Information). Surprisingly, under high compression, HeLa cells increased the adhesion strengthening rate to BSA ≈6.3‐fold, while fibroblasts increased the adhesion strengthening rate to FNIII7‐10ΔRGD ≈3.3‐fold.

The findings suggest that with increasing compression, cells accelerate the adhesion strengthening to ECM proteins, which does not arise from increasing the contact area. However, at high compression, the cells also drastically accelerate the unspecific adhesion strengthening.

### 2.3 Unspecific Cell‐ECM Adhesion Increases Considerably at High Compression

**Figure 2:**
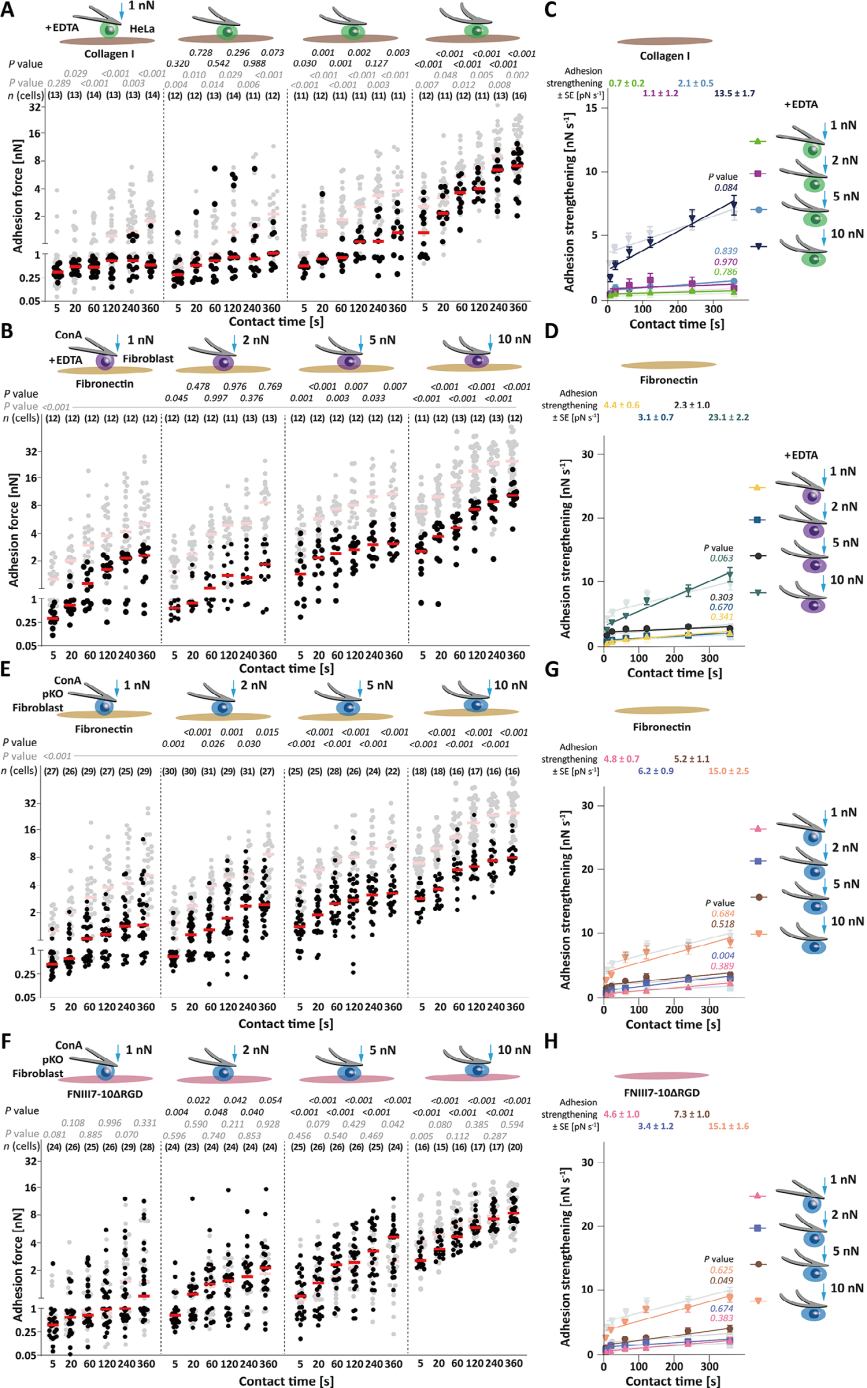
Under high compression force, unspecific adhesion considerably contributes to cell‐ECM adhesion. A,B) Adhesion force of A) EDTA‐treated HeLa cells to collagen I or B) EDTA‐treated fibroblasts to fibronectin at 1, 2, 5, and 10 nN compression force. Dots represent adhesion forces of single cells, red bars median values, and *n*(cells) the number of independent cells tested in at least three independent experiments. Adhesion force under given compression force of untreated A) HeLa cells to collagen I or B) of untreated fibroblasts to fibronectin is given as reference (semitransparent; data taken from Figure 1A,B). *p* values in gray compare adhesion force of given and reference data. *p* values in black compare adhesion force of the displayed data with the adhesion force of EDTA‐treated A) HeLa cells to collagen I or B) fibroblasts to fibronectin at 1 nN compression force. *p* values were calculated by two sided Mann–Whitney tests. Statistical analysis comparing the adhesion force of the displayed data with untreated A) HeLa cells to BSA or B) fibroblasts to FNIII7‐10ΔRGD is given in Table S4 (Supporting Information). C,D) Adhesion strengthening rate of C) EDTA‐treated HeLa cells to collagen I and D) EDTA‐treated fibroblasts to fibronectin as quantified by the slope of a linear regression fit of adhesion force to unrestricted ECM protein for all contact times. Values give best fit values of adhesion strengthening rates ± SE. Dots depict mean adhesion forces, error bars SEM, and lines linear regressions. Adhesion strengthening rates of C) untreated HeLa cell to BSA and D) fibroblasts to FNIII7‐10ΔRGD are given in the background (semitransparent; data taken from Figure 1G,H). *P* values comparing adhesion strengthening rates with reference data were calculated by extra sum squares *F* test. E–H) Adhesion force of pKO fibroblasts to E) fibronectin or F) FNIII7‐10ΔRGD and adhesion strengthening rate of pKO fibroblasts to G) fibronectin or H) FNIII7‐10ΔRGD under given compression forces. Adhesion force of wild‐type fibroblasts to E) fibronectin or F) FNIII7‐10ΔRGD and adhesion strengthening rate of wild‐type fibroblasts to G,H) FNIII7‐10ΔRGD are given as reference in the background (semitransparent; data taken from Figure 1 B,D,H). E,F) *p* values in gray compare adhesion force of given and reference data. *p* values in black compare adhesion force of the displayed data with the adhesion force of pKO fibroblasts to E) fibronectin or F) FNIII7‐10ΔRGD at 1 nN compression force. *p* values comparing adhesion forces of given and reference data were calculated by two sided Mann‐Whitney tests. G, H) *P* values comparing given adhesion strengthening rates with reference data were calculated by extra sum squares *F* test.

To test whether at high compression the unspecific adhesion contributes to the cell‐ECM adhesion, we deprived HeLa cells and fibroblasts from divalent ions by ethylenediaminetetraacetic acid (EDTA) chelation, which curbs integrin‐ligand binding,^[^ ^39^ ^]^ and tested their adhesion to collagen I or fibronectin at different compression forces (**Figure** **2**A,B). As expected, divalent ion‐deprivation drastically reduced the adhesion force of HeLa cells to collagen I and of fibroblasts to fibronectin for all compression forces. Under low compression, the adhesion force of EDTA‐treated HeLa cells to collagen I and of EDTA‐treated fibroblasts to fibronectin was very low and unaffected by the compression force applied (1 or 2 nN). Under medium compression, both cell lines slightly increased the adhesion force. However, under high compression both cell lines drastically increased the adhesion force, although the addition of EDTA prevented integrins from binding ECM proteins. The adhesion strengthening rates of EDTA‐treated HeLa cells to collagen I or of fibroblasts to fibronectin were similar at low and medium compression (Figure 2C,D). However, at high compression, the adhesion strengthening of EDTA‐treated HeLa cells to collagen I increased ≈6.5‐fold and of EDTA‐treated fibroblasts to fibronectin ≈5.3‐fold. Thus, the results suggest that under high compression unspecific adhesion mechanisms contribute considerably to cell‐ECM adhesion.

To further test this hypothesis, we quantified the adhesion force of pan‐integrin deficient (pKO) fibroblasts^[^ ^40^ ^]^ to fibronectin and FNIII7‐10ΔRGD at low,Tmedium, and high compression (Figure 2E,F). pKO fibroblasts established similar adhesion force to fibronectin and FNIII7‐10ΔRGD for each compression force. At high compression, pKO fibroblasts established up to ≈5‐fold higher unspecific adhesion force to fibronectin and FNIII7‐10ΔRGD compared to low compression. Further, pKO fibroblasts strengthened adhesion to fibronectin and FNIII7‐10ΔRGD in the absence of integrins similarly to wt fibroblasts to FNIII7‐10ΔRGD (Figure 2G,H). Therefore, fibroblasts subjected to high compression employ unspecific adhesion mechanisms to increase cell adhesion to fibronectin and accelerate adhesion strengthening.

In summary, under high compression, HeLa cells and fibroblasts establish integrin‐mediated and unspecific adhesion to collagen I or fibronectin. Thereby, at high compression the unspecific cell adhesion can approach the values measured for integrin‐mediated cell adhesion at lower compression. Hence, under high compression, cells must employ other cell surface molecules in addition to integrins to adhere to ECM proteins.

### 2.4 Under High Compression Cells Predominantly Establish Unspecific Adhesion

**Figure 3:**
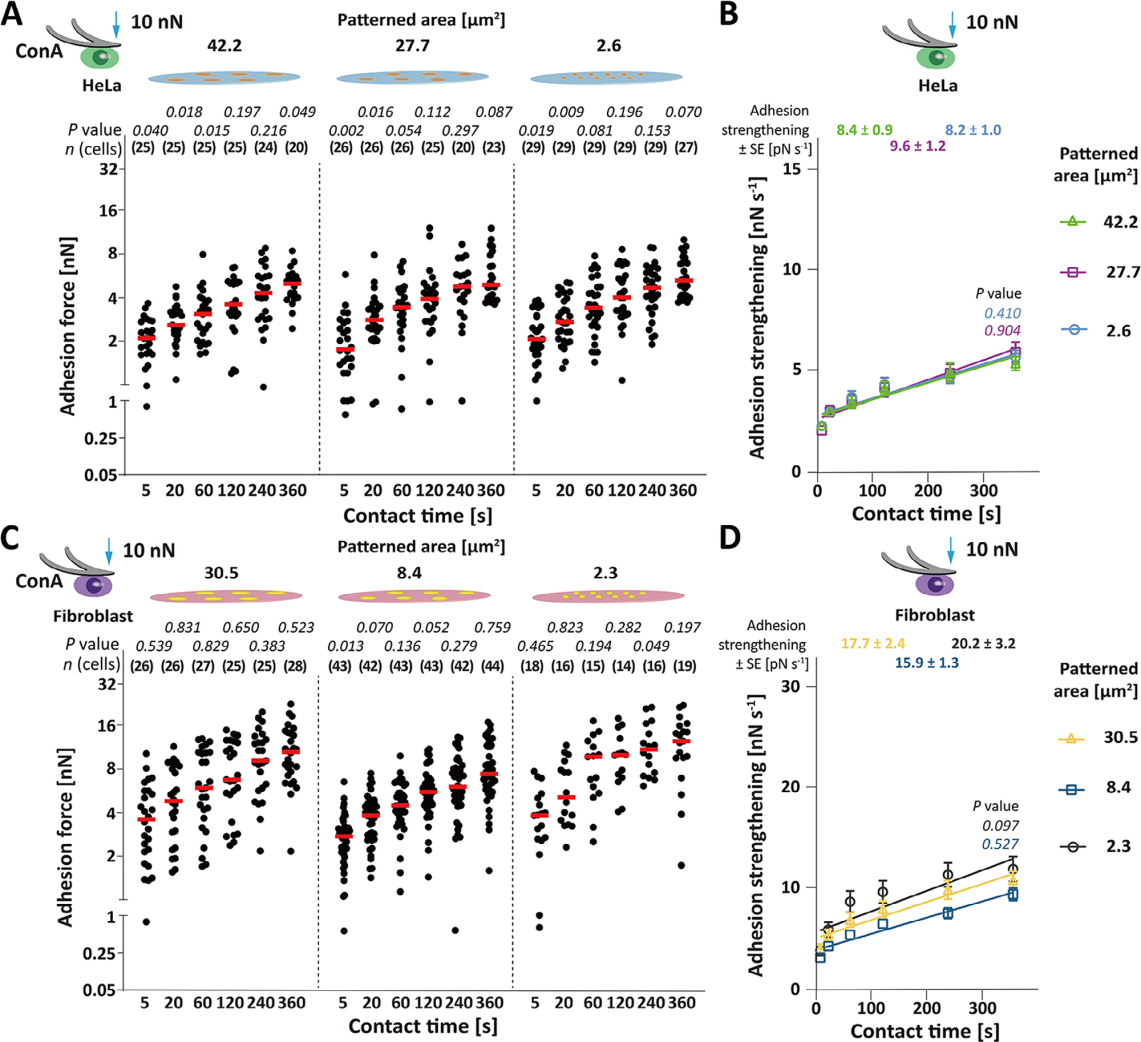
Unspecific adhesion dominates adhesion to mixed substrates at 10 nN compression force. A,C) Adhesion force of A) HeLa cells and C) fibroblasts at 10 nN compression force to substrates with ECM protein patterns of given areas. Glass surfaces were coated with BSA for collagen I patterns and with FNIII7‐10ΔRGD for fibronectin patterns. Dots represent adhesion forces of single cells, red bars median values, and *n*(cells) the number of independent cells tested in at least three independent experiments. *p* values compare the adhesion force on indicated patterns to adhesion forces of A) HeLa cells to unrestricted BSA, of C) fibroblasts to unrestricted FNIII7‐10ΔRGD substrate at 10 nN compression force. *p* values were calculated by two sided Mann–Whitney tests. Statistical analysis of adhesion force established by HeLa cells to collagen I patterns and fibroblasts to fibronectin patterns of different sizes is given in Tables S5 and S6 (Supporting Information). B,D) Adhesion strengthening rate of B) HeLa cells to collagen I patterns and D) fibroblasts to fibronectin patterns as quantified by the slope of a linear regression fit of the cell adhesion force (data taken from Figure 3A,C). Values give best fit values of adhesion strengthening rates ± SE. Dots depict mean adhesion forces, error bars SEM, and lines linear regressions. *p* values were calculated by extra sum squares *F* test and compare the slopes at the indicated compression force with the slope of B) HeLa cells to collagen I patterns having areas of ≈ 42.2 µm^2^ or of D) fibroblasts to fibronectin patterns having areas of ≈ 30.5 µm^2^.

Next, we aimed to understand the contribution of integrin‐mediated and unspecific adhesion to initiate cell adhesion under high compression. Thereto, we microcontact printed circular collagen I or fibronectin patterns of different sizes on glass. The printed collagen I patterns covered areas of 42.2 ± 8.0 µm^2^ (mean ± SD), 27.7 ± 7.5 µm^2^, and 2.6 ± 1.0 µm^2^ and the fibronectin patterns covered areas of 30.5 ± 4.8, 8.4 ± 2.1, and 2.3 ± 2.1 µm^2^ (Figure S4, Supporting Information). After printing, we coated the uncoated glass surface surrounding the printed collagen I patterns with BSA, while the uncoated glass surface surrounding the printed fibronectin patterns was coated with FNIII7‐10ΔRGD (Figure S5A, Supporting Information). Then we measured the adhesion force of HeLa cells and fibroblasts to the ECM protein patterns under low and high compression. The contact area between cell and substrate was always larger than the area of the printed ECM protein pattern, which allowed the cells to establish adhesion to the ECM proteins and unspecific substrates (Figure S5B, Supporting Information). To understand whether cells were able to establish integrin‐mediated adhesion to ECM protein patterns at different compression forces, we localized paxillin of paxillin‐GFP expressing HeLa cells and fibroblasts using timelapse microscopy (Figures S6 and S7, Supporting Information). The results show that both cell lines accumulated paxillin at the circumference of the ECM protein patterns within 10 min, independent of the pattern size and compression force. This accumulation of paxillin at the circumference of the ECM protein patterns demonstrated that both cell lines were able to establish integrin‐mediated adhesion to the ECM protein patterns. SCFS experiments showed that while at low compression the adhesion force and adhesion strengthening of HeLa cells depends on the size of the collagen I pattern, at high compression the cell adhesion force and adhesion strengthening were independent of the collagen I pattern size (**Figure** **3**A,B; Figure S8A,B, Table S5, Supporting Information). Further, at low compression the cell adhesion force to collagen I patterned substrates was higher compared to unrestricted BSA substrates, while at high compression the HeLa cells established either similar or slightly lower adhesion forces to patterned collagen I substrates compared to unrestricted BSA substrates. Similarly, while under low compression fibroblasts established fibronectin pattern size dependent adhesion force, under high compression, the adhesion force and strengthening were independent of the fibronectin pattern size and mostly similar to those measured to unrestricted FNIII7‐10ΔRGD substrate (Figure 3C,D; Figure S8C,D, Table S6, Supporting Information). Since these results indicate that adhesion force of fibroblasts to fibronectin patterns is integrin‐independent, we quantified the adhesion force of pKO fibroblasts, which express no fibronectin‐binding integrins, to fibronectin patterns under low and high compression (Figure S8E,F, Supporting Information). The results show that pKO fibroblasts adhere very weakly under low compression to fibronectin patterns with the adhesion force being much below that established by wt fibroblasts. Hence, under low compression, the adhesion force of wt fibroblasts to fibronectin patterns depends on fibronectin‐binding integrins. Under high compression, however, pKO fibroblasts established high adhesion force to fibronectin patterns, which is, as expected, independent of the fibronectin pattern size. Thereby, under high compression pKO and wt fibroblasts established similarly high adhesion force to fibronectin patterns.

In summary, while under low compression the adhesion force of HeLa cells and fibroblasts depends on the size of the ECM protein pattern, under high compression the adhesion force neither depends on the area of the ECM protein pattern nor on the presence of integrins. These results strongly argue that under high compression the unspecific adhesion is independent of the presence of integrins and that this unspecific adhesion is the major contributor to the adhesion force the cells establish to ECM protein patterns.

### 2.5 Glycocalyx Mediates Unspecific Cell Adhesion and Regulates Integrin‐Mediated Cell Adhesion

To understand how cells increase early adhesion in response to compression, we tested the involvement of the glycocalyx. Prior to SCFS, we digested the glycocalyx of HeLa cells and fibroblasts 3 h using a glycosidase cocktail. We evaluated the efficacy of glycocalyx digestion by incubating the glycosidase cocktail‐treated cells with fluorescently labeled lectin and analyzing their fluorescent intensity by flow cytometry (Figure S9A,B, Supporting Information). As positive control we used untreated cells incubated with fluorescently labeled lectin and as negative control we used unlabeled cells. The efficiency of glycocalyx removal was ≈75% for glycosidase‐treated HeLa cells and ≈84% for fibroblasts. Digesting the glycocalyx neither affected the surface expression levels of β1‐class integrins nor their conformational state in rounded HeLa cells and fibroblasts (Figure S9C–F, Supporting Information). For SCFS, we coated the microcantilevers with Cell‐tak to firmly attach the glycocalyx‐digested cells. Control experiments showed that attaching HeLa cells or fibroblasts to Cell‐tak‐coated microcantilevers affected neither their adhesion force to collagen I or fibronectin nor their unspecific adhesion to BSA or FNIII7‐10ΔRGD (Figure S10, Supporting Information).

**Figure 4:**
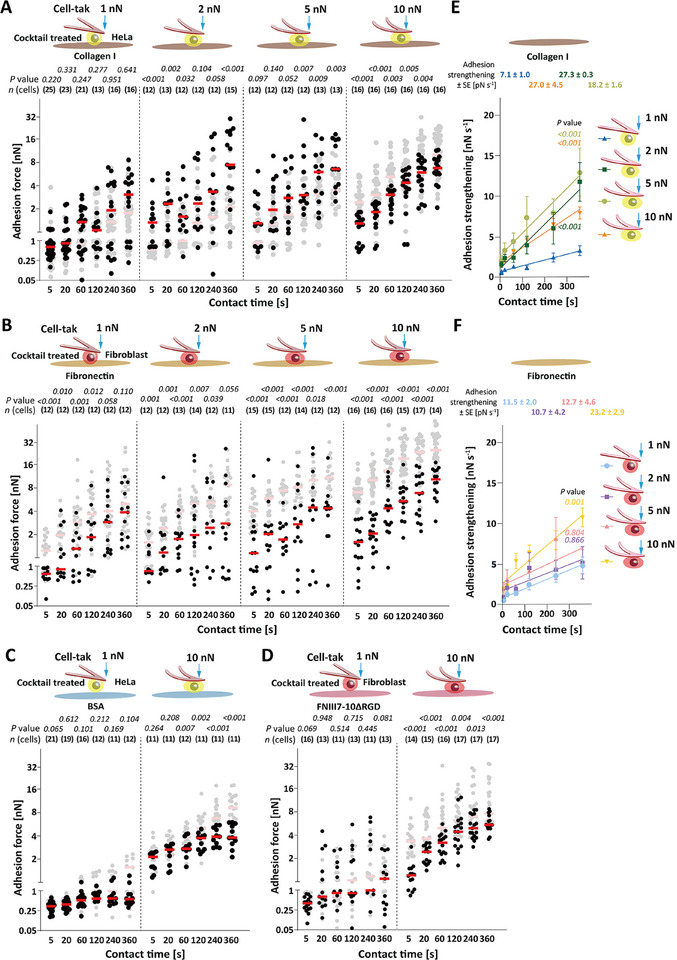
The glycocalyx facilitates unspecific adhesion and modulates integrin‐mediated adhesion. A–D) Adhesion force of glycosidase cocktail‐treated HeLa cells to A) collagen I or B) BSA and of fibroblasts to C) fibronectin or D) FNIII7‐10ΔRGD substrates at given compression forces. Cells were attached to Cell‐tak‐coated cantilevers. Dots represent adhesion forces of single cells, red bars median values, and *n*(cells) the number of independent cells tested in at least three independent experiments. Adhesion force of A) wild‐type (wt)HeLa cells attached to ConA‐coated cantilevers to collagen I or C) wt fibroblasts to fibronectin and B) wt HeLa attached to Cell‐tak‐coated cantilevers cells to BSA or D) wt fibroblasts to FNIII7‐10ΔRGD is given as reference (semitransparent). *p* values were calculated by two sided Mann–Whitney tests and compare displayed and reference adhesion force. Statistical analysis of adhesion force established by glycosidases cocktail‐treated HeLa cells to collagen I and fibroblasts to fibronectin of different compression force is given in Tables S7 and S8 (Supporting Information). E,F) Adhesion strengthening rate of E) glycosidase‐cocktail treated HeLa cells to collagen I and F) glycosidase‐cocktail treated fibroblasts to fibronectin. Adhesion strengthening rates were quantified as the slope of a linear regression fit of cell adhesion force to E) collagen I or F) fibronectin for all contact times under the given compression force. Dots depict means, error bars SEM, and lines linear regressions. *p* values were calculated by extra sum‐of‐squares *F* test and compare the slopes at the indicated compression force with the one at 1 nN.

Next, we quantified the adhesion force of glycocalyx‐digested HeLa cells to collagen I and glycocalyx‐digested fibroblasts to fibronectin under low to high compression (**Figure** **4**A). Glycocalyx digestion for 3 h did not affect the adhesion force of HeLa cells to collagen I at 1 nN compression. At 2 and 5 nN compression 3 h glycocalyx‐digested HeLa cells established higher adhesion force to collagen I compared to untreated HeLa cells. In contrast, at 10 nN compression, the glycocalyx digestion reduced the adhesion force of HeLa cells to collagen I. Interestingly, the adhesion force and adhesion strengthening of glycocalyx‐digested HeLa cells to collagen I under 2, 5, and 10 nN compression were similar and only slightly higher than at 1 nN compression (Figure 4A,E). Importantly, glycocalyx digestion did not affect the adhesion of HeLa cells to BSA at 1 nN compression but substantially reduced the adhesion force of HeLa cells to BSA at 10 nN compression and contact times >20 s (Figure 4C). In fibroblasts, glycocalyx digestion decreased the adhesion force to fibronectin and FNIII7‐10ΔRGD for all compression forces (Figure 4B,D). The compression force dependency of the adhesion initiation of fibroblasts greatly reduced, whereas the adhesion force and adhesion strengthening rate increased only at 10 nN compression compared to 1, 2, and 5 nN compression (Figure 4F).

To show the specificity of the glycosidase cocktail treatment, we incubated HeLa cells and fibroblasts for 1 h with the glycosidase cocktail. Flow cytometry showed that 1 h treatment with the glycosidase cocktail removed ≈14% of the glycocalyx of HeLa cells (compared to ≈75% removal after 3 h treatment) and removed ≈25% of the glycocalyx of fibroblasts (compared to ≈85% removal after 3 h treatment, Figure S9A,B, Supporting Information). We next quantified the adhesion force of 1 h glycosidase cocktail‐treated HeLa cells to BSA and fibroblasts to FNIII7‐10ΔRGD by SCFS (Figure S11A,B, Supporting Information). The results show that the unspecific adhesion force of HeLa cells and fibroblasts ranged between the untreated and the 3 h glycosidase cocktail‐treated cells. Furthermore, the cell adhesion force exponentially decayed with the increasing removal of the glycocalyx (Figure S11C,D, Supporting Information). These results argue for a contribution of the glycocalyx to the unspecific adhesion of cells.

In summary, the results show that the glycocalyx mediates unspecific adhesion of HeLa cells and fibroblasts and regulates the compression dependence of integrin‐mediated adhesion to collagen I or fibronectin.

### 2.6 Unspecific Cell Adhesion is Independent of Integrin Activation and Actin Engagement

**Figure 5:**
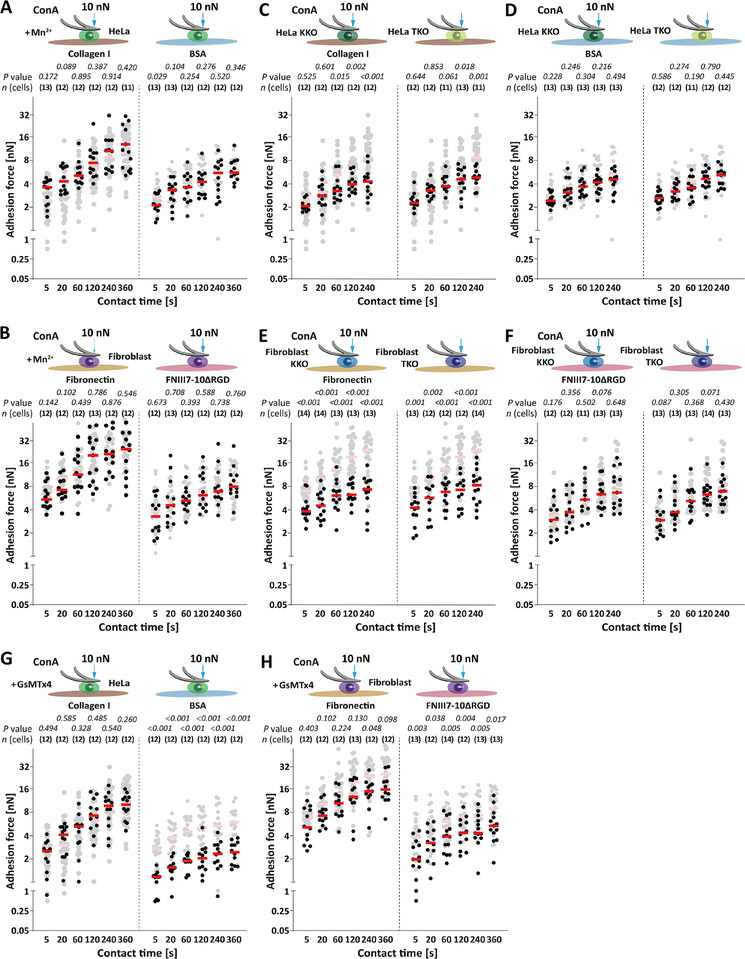
At high compression, unspecific adhesion is independent of integrin pre‐activation and talin/kindlin but depends on mechanosensitive ion channels. A, B) Adhesion force of A) 0.5 mm Mn^2+^‐treated HeLa cells to collagen I or BSA and B) of 0.5 mm Mn^2+^‐treated fibroblasts to fibronectin or FNIII7‐10ΔRGD. The adhesion force of untreated cells is given in the background as reference (semitransparent). Reference data taken from Figure 4C,D. (C,D) Adhesion force of kindlin‐depleted (KKO) HeLa cells and talin‐depleted (TKO) HeLa cells to C) collagen I or D) BSA at given contact times. E,F) Adhesion force of kindlin‐depleted (KKO) fibroblasts and talin‐depleted (TKO) fibroblasts to E) fibronectin or F) FNIII7‐10ΔRGD at given contact times. Adhesion force of wt HeLa cells or wt fibroblasts in the respective condition are given as reference (semitransparent). Reference data taken from Figure 4A–D. (G,H) Adhesion force of 10 µm GsMTx4‐treated G) HeLa cells to collagen I or BSA and of 10 µm GsMTx4‐treated H) fibroblasts to fibronectin or FNIII7‐10ΔRGD at high compression force of 10 nN and given contact times. Adhesion force of untreated HeLa cells or fibroblasts in the respective condition are given as reference (semitransparent). Reference data taken from Figure 4A–D. A–H) Dots represent adhesion forces of individual cells, red bars the median, and *n* (cells) the number of individual cells tested in at least three independent experiments. *p* values were calculated by two sided Mann–Whitney tests and compared the adhesion forces of given data with the reference data.

Next, we aimed at understanding whether integrin activation and their connection to the actomyosin cortex is required for the compression induced cell adhesion. First, we incubated rounded HeLa cells and fibroblasts 30 min prior to and throughout the SCFS experiments with 0.5 mm Mn^2+^, which induces an extended conformation of integrins.^[^ ^41^, ^42^ ^]^ We approached Mn^2+^‐treated HeLa cells to collagen I‐ or BSA‐coated substrates and Mn^2+^‐treated fibroblasts to fibronectin‐ or FNIII7‐10ΔRGD‐coated substrates until reaching a high compression force of 10 nN (**Figure** **5**A,B). The pre‐activation of integrins did not affect the adhesion of HeLa cells to collagen I or BSA nor of fibroblasts to fibronectin or FNIII7‐10ΔRGD. These findings indicate that under high compression cell adhesion to specific and unspecific substrates is not regulated by the transition of integrins into the active conformation.

Further, we investigated how the two major adaptor proteins, kindlin and talin, involve in the compression‐dependent integrin‐mediated and unspecific adhesion. Previously, we showed that under low compression the integrin‐mediated adhesion to the ECM depends on the expression of both adaptor proteins.^[^ ^43^, ^44^ ^]^ In these experiments, the depletion of talin or kindlin in HeLa cells and fibroblasts reduced the adhesion force to ECM proteins to very low levels similar to unspecific adhesion. Hence, we quantified the adhesion force of kindlin‐depleted (KKO) or talin‐depleted (TKO) HeLa cells to collagen I or BSA and of KKO or TKO fibroblasts to fibronectin or FNIII7‐10ΔRGD under high compression (Figure 5C–F). While the depletion of talin and kindlin drastically reduced the adhesion force of HeLa cells to collagen I and of fibroblasts to fibronectin, the adhesion of these cells to BSA or FNIII7‐10ΔRGD remained unaffected. Importantly, the adhesion force of KKO and TKO HeLa cells to collagen I and BSA as well as of KKO and TKO fibroblasts to fibronectin and to FNIII7‐10ΔRGD were similar, which indicates that their adhesion force was mainly mediated by the glycocalyx and/or components of the cell surface, other than integrin. Hence, the results show that intracellular integrin activation and their connection to the actomyosin cortex via talin and kindlin is important to establish specific integrin‐mediated adhesion ECM proteins under high compression. However, both adaptor proteins do not contribute to the compression induced unspecific adhesion to ECM proteins or other proteins cells commonly do not adhere to.

Together, these findings indicate that under high compression cell adhesion to ECM proteins and unspecific substrates is not regulated by the transition of integrins into the active conformation. Further, the results show that intracellular integrin activation and their connection to the actomyosin cortex via talin and kindlin is important to establish integrin‐mediated adhesion ECM proteins under high compression. However, both adaptor proteins do not contribute to the compression induced unspecific adhesion to ECM proteins or proteins cells commonly do not adhere to.

### 2.7 Unspecific Cell Adhesion at High Compression Depends on Mechanosensitive Ion Channels

Finally, we investigated the involvement of mechanosensitive ion channels on the observed strengthening of the unspecific adhesion of compressed cells. To this end we incubated suspended HeLa cells and fibroblasts with 10 µm GsMTx4,^[^ ^45^ ^]^ which inhibits a broad spectrum of mechanosensitive ion channels, 30 min prior to and throughout the SCFS experiment (Figure 5G,H). We quantified the adhesion force of GsMTx4‐treated HeLa cells to collagen I or BSA as well as of GsMTx4‐treated fibroblasts to fibronectin or FNIII7‐10ΔRGD under high compression (10 nN). GsMTx4 treatment did not affect the adhesion force of HeLa cells to collagen I (Figure 5G). However, GsMTx4 treatment strongly reduced the unspecific adhesion force of HeLa cells to BSA at all contact times. Interestingly, the unspecific adhesion force was even lower than that observed for 3 h glycocalyx‐digested HeLa cells. GsMTx4‐treated fibroblasts tentatively established lower adhesion force to fibronectin under high compression, though this was statistically significant only at 240 s contact time (Figure 5H). However, GsMTx4‐treated fibroblasts established considerably lower unspecific adhesion force to FNIII7‐10ΔRGD at all contact times. Compared to 3 h glycocalyx‐digested fibroblasts, the unspecific adhesion force of GsMTx4‐treated fibroblasts to FNIII7‐10ΔRGD was slightly higher at < 60 s and similar at ≥ 60 s contact time. This shows a similar but distinct response of fibroblasts to GsMTx4 compared to HeLa cells.

Together, these results suggest that the compression‐induced unspecific adhesion of HeLa cells and fibroblasts to BSA or FNIII7‐10ΔRGD involves mechanosensitive ion channels.

## 3 Conclusion

**Figure 6:**
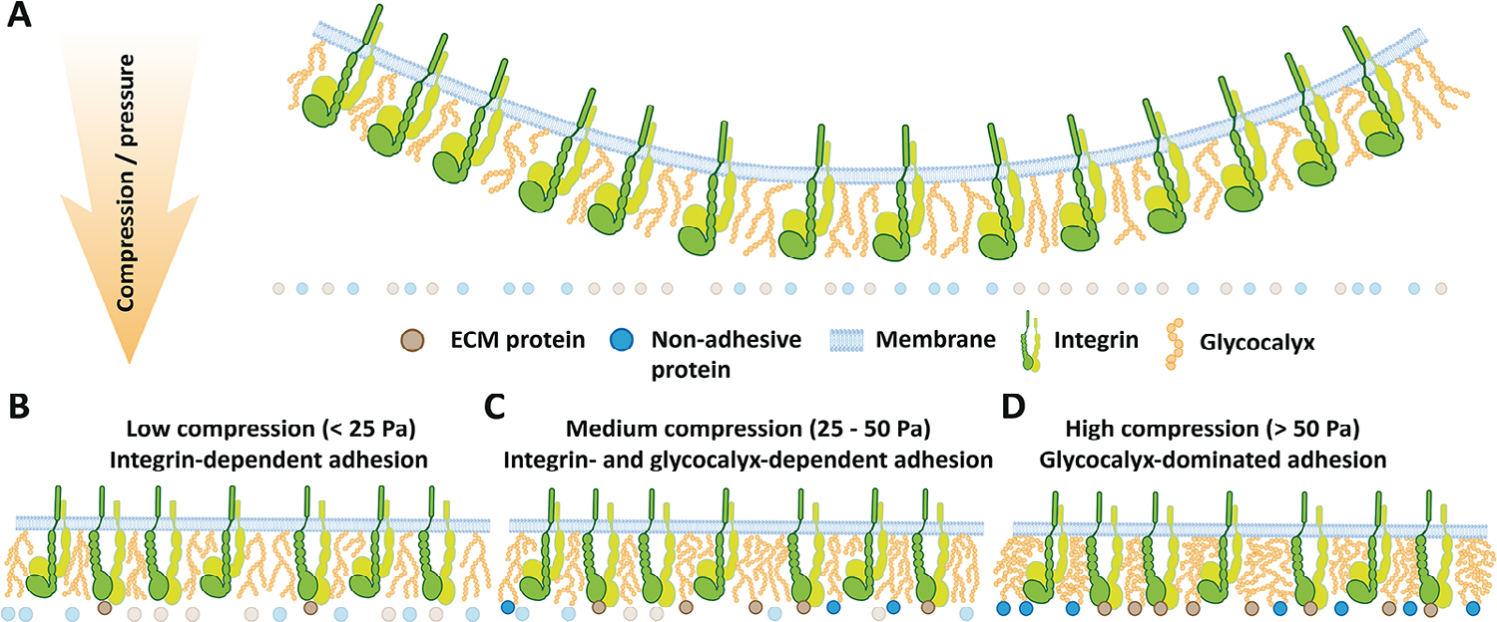
Glycocalyx synergizes with integrin to mediate cell adhesion under compression. A) Normal state of membrane with bent‐closed integrins surrounded by glycocalyx. B) Integrins mediate specific cell adhesion to ECM proteins under low compression, while the glycocalyx contributes weak unspecific adhesion. C) Under medium compression, the unspecific adhesion the glycocalyx contributes to the overall cell adhesion increases. D) Under high compression, the unspecific adhesion of the glycocalyx to ECM and non‐ECM proteins contributes significantly to, and can even dominate, the overall cell adhesion.

Here, we investigate how compression triggers mammalian cells to regulate the initiation and strengthening of integrin‐mediated adhesion to ECM proteins and unspecific adhesion to other proteins. We apply low (1 and 2 nN compression force), medium (5 nN), and high (10 nN) cellular compression in SCFS before quantifying the cell adhesion force (**Figure** **6**A). Increasing the compression increases the contact area between cell and substrate and the externally applied pressure to the cell. The compression pressures applied in our experiments are < 80 Pa and do not exceed the intracellular pressure (10 – 3000 Pa) typically generated by animal cells.^[^ ^46^, ^47^, ^48^, ^49^ ^]^ While increasing from low to high compression increases the contact area between cells and ECM substrate ≈1.9‐fold for HeLa cells and ≈1.4‐fold for fibroblasts, it increases the adhesion strengthening rate of HeLa cells to collagen I ≈6.8‐fold and of fibroblasts to fibronectin ≈3.1‐fold, thus showing that cells subjected to compression considerably strengthen and accelerate adhesion initiation to the ECM (Figure 6B–D). We further confirm previous studies showing that the adhesion of HeLa cells to BSA and of fibroblasts to FNIII7‐10ΔRGD is minimal for low compression.^[^ ^43^, ^50^, ^51^, ^52^, ^53^ ^]^ However, subjected to high compression, HeLa cells and fibroblasts drastically increase the adhesion force and accelerate adhesion strengthening to BSA and FNIII7‐10ΔRGD to which integrins do not bind. Importantly, we show that this unspecific adhesion neither depends on integrin activation, nor on the adaptor proteins kindlin and talin that maintain integrins in the active conformation and engage integrins to the actomyosin cortex. The threshold compression at which cells increase adhesion seems to be cell type dependent. Although rounded HeLa cells considerably increase adhesion force and strengthen adhesion faster if compressed by > 15% of the cell height, fibroblasts already increase adhesion if compressed by > 10%.

Interestingly, our results also show that cells under high compression can establish strong integrin‐mediated cell adhesion and strong unspecific cell adhesion simultaneously. To understand whether and how integrin‐mediated and unspecific adhesion cooperate under compression, we used circular ECM protein patterns of different sizes that are surrounded by non‐adhesive proteins. The results show that cells establish mainly integrin‐mediated adhesion to ECM proteins under low compression. However, with increasing compression, the cells considerably increase the unspecific adhesion force and strengthen the unspecific adhesion force faster. Finally, at high compression, unspecific adhesion contributes more to the cell adhesion force to ECM patterned surfaces compared to integrin‐mediated adhesion.

In the search for the mechanism involved in the switch from low to high unspecific cell adhesion, we find that the glycocalyx drives cell adhesion under high compression. Partial removal of the glycocalyx does not affect the unspecific adhesion of HeLa cells or fibroblasts at low compression.^[^ ^54^, ^55^, ^56^, ^57^ ^]^ Together with a report showing cells reduced cell adhesion to BSA after ≈95% removal of glycocalyx,^[^ ^34^ ^]^ our results indicate that a relatively small fraction of glycosylation on the cell surface (≈25% for HeLa cells and ≈15% for fibroblasts) is sufficient to establish low unspecific cell adhesion. Indeed, we show an exponential decay of the adhesion force with increasing glycocalyx removal. However, the high unspecific cell adhesion in response to high compression depends on an intact glycocalyx of fibroblasts and HeLa cells. Our analysis indicates that highly compressed cells can establish unspecific adhesion even with completely removed glycocalyx, which suggests other contributors to the unspecific adhesion of cells. Notably, while our experiments indicate that the glycocalyx has no effect on integrin conformation regulation, we find opposing roles of the glycocalyx in HeLa cells and fibroblasts in establishing integrin‐mediated adhesion. Although in HeLa cells, glycocalyx digestion does not affect adhesion initiation at low compression, it increases the adhesion force to collagen I at medium compression. On the contrary, glycocalyx digestion of fibroblasts considerably reduces the adhesion force to fibronectin at low and medium compression. These results suggest that the glycocalyx plays an inhibitory role in HeLa cells adhering to collagen I and promotes the adhesion of fibroblasts to fibronectin. The different roles of the glycocalyx in cell‐ECM adhesion could arise from different glycosylation of cancer cells, such as HeLa cells, compared to non‐transformed cells, such as fibroblasts.^[^ ^58^, ^59^, ^60^ ^]^ The glycocalyx of cancer cells is much thicker and denser than in non‐transformed cells due to the overexpression of mucins.^[^ ^60^, ^61^ ^]^ The thickness of the glycocalyx ranges from tens to hundreds of nanometers, depending on cell type and cell state, and hence largely exceeds the ≈19 nm integrins extrude from the cell membrane when they adopt an extended conformation.^[^ ^62^, ^63^ ^]^ Hence, we hypothesize that a thicker glycocalyx shields integrins from binding to the ECM proteins. Consistently it was demonstrated that the overexpressed glycoprotein mucin in cancer cells, with longer glycosylation chains, reduces the integrin binding rate of cancer cells and enhances integrin clustering and focal adhesion formation during adhesion maturation in a kinetic trap‐like manner.^[^ ^32^, ^33^ ^]^ We thus propose that the thick and dense glycocalyx hinders integrin binding on HeLa cells to collagen I at low and medium compression, and that the partial removal of the glycocalyx increases integrin binding to collagen I due to the reduced physical barrier formed by glycocalyx. Differently, under high compression the glycocalyx considerably contributes to the adhesion force of HeLa cell to collagen I. We propose that the observed reduction of adhesion force to collagen I at high compression is due to a strong reduction of the glycocalyx mediated adhesion, which may be partially compensated by an increased integrin mediated adhesion as found for low and medium compression. These two opposing effects of glycocalyx digestion results in a net reduction of the cell adhesion force measured at high compression. However, such adhesive roles may also depend on the cell‐type specific composition of the glycocalyx.

In vivo, cells are commonly confined by their environment, which regulates their migratory behavior.^[^ ^12^, ^64^ ^]^ Our experiments suggest that with increasing compression cells increases the unspecific adhesion to low adhesive surfaces, such as it would be necessary for the cell to switch to the amoeboid‐like migration mode on minimal‐adhesive surfaces.^[^ ^1^, ^2^, ^4^, ^65^ ^]^ Interestingly, mechanosensitive ion channels have been linked to confined cell migration.^[^ ^66^, ^67^, ^68^, ^69^ ^]^ We show here that while integrin‐mediated adhesion initiation of highly compression of HeLa cells and fibroblasts is independent of mechanosensitive ion channels, unspecific cell adhesion to minimally adhesive substrates involves mechanosensitive ion channels under high compression. Thereby, the inhibition of a wide spectrum of mechanosensitive ion channels reduces the unspecific adhesion force under high compression to similar or even lower levels as the removal of the glycocalyx. This indicates that mechanosensitive ion channels may play a major role in amoeboid‐like migration of confined cells by regulating friction generating adhesion mechanisms.^[^ ^2^, ^28^, ^29^, ^30^ ^]^ However, which mechanosensitive ion channels involve in the compression‐dependent adhesion strengthening and how they involve in regulating the unspecific cell adhesion response remains to be shown.

## 4 Experimental Section

### Cell Culture

Wild‐type HeLa (Kyoto) (kind gift from A. Hyman, MPI Molecular Cell Biology and Genetics, Germany), talin1/2‐depleted (TKO) HeLa cells,^[^ ^43^ ^]^ kindlin1/2‐depleted (KKO) HeLa cells,^[^ ^43^ ^]^ wt HeLa cells over expressing paxillin‐GFP, pan‐integrin deficient fibroblasts (pKO),^[^ ^40^ ^]^ and wild‐type mouse embryonic kidney fibroblasts,^[^ ^40^ ^]^ TKO fibroblasts,^[^ ^44^ ^]^ KKO fibroblasts,^[^ ^44^ ^]^ wt fibroblasts over expressing paxillin‐GFP were cultured in Dulbecco's modified eagle medium (DMEM, 31 966 047, Thermo Fisher Scientific), supplemented with 10% (vol/vol) fetal bovine serum (FBS, F9665, Sigma–Aldrich), 100 U mL^−1^ penicillin and 100 µg mL^−1^ streptomycin (15 140 122, Thermo Fisher Scientific). To visualize paxillin‐GFP, wt HeLa cells and wt fibroblasts were transfected with plasmids encoding for paxillin‐GFP (#50 529, Addgene) using lipofectamine 2000 (11 668 027, Thermo Fisher Scientific) following the protocol provided by the manufacturer.

### Cantilever Functionalization and Cell Attachment

Tipless microcantilevers with a nominal spring constant of 0.06 N m^−1^ (NP‐O, Bruker) were plasma cleaned (PDC‐32G, Harrick Plasma) for 5 min and incubated overnight with 2 mg mL^−1^ concanavalin A (ConA, C2010‐100MG, Sigma–Aldrich) in PBS or 20 µg mL^−1^ Cell‐tak (Corning Cell‐Tak Zell‐ und Gewebekleber, 354 240, Corning) in 16.7 mm NaOH and 0.9 mm NaHCO_3_, pH 8.0 at 4 °C. Cells were grown in 12‐well plates to a maximal confluency of ≈80%. Before experiments, cells were serum‐starved for at least 1 h before single‐cell force spectroscopy (SCFS). Then cells were washed with PBS and detached with 200 µL of 0.25% (wt/vol) trypsin/EDTA (25 200 072, Thermo Fisher Scientific) for 2 min at 37 °C. Detached cells were suspended in 1% (vol/vol) FCS containing DMEM (12 800 017, Thermo Fisher Scientific) supplemented with 20 mm HEPES (SCFS medium). Cells were pelleted and resuspended in 200 µL FCS‐free SCFS medium. After detachment, cells were allowed to recover for 30 min from trypsin/EDTA treatment in SCFS medium at 37 °C.^[^ ^53^ ^]^ For integrin inactivation experiments, 10 mM EDTA was added into the SCFS medium 30 min prior to the experiments and throughout the adhesion measurements. For SCFS in the presence of Mn^2+^ or GsMTx4, suspended cells were incubated with 0.5 mm MnCl_2_ (Manganese (II) chloride solution, M1787, Sigma–Aldrich) or 10 µm GsMTx4 (HY‐P1410, MedChemExpress) in SCFS medium for at least 30 min and the chemicals were present throughout the experiments.

### Single‐Cell Force Spectroscopy (SCFS)

SCFS was performed using an AFM‐based CellHesion 200 or an AFM (NanoWizard II) equipped with a CellHesion‐module (all JPK instruments) mounted on an inverted microscope (AxioObserver, Zeiss). The ambient temperature was maintained at 37 °C by a PetriDish‐Heater (JPK instruments). The exact spring constant of each used cantilever was calibrated prior to experiments using the thermal noise method. To attach a single cell to the ConA‐ or Cell‐tak‐coated cantilever, suspended single cells were pipetted onto BSA or FNIII7‐10ΔRGD‐coated areas of Petri dishes. The cantilever was lowered onto a single cell with 10 µm s^−1^ until detecting a force of 5 nN. After 5 s contact time the cantilever was retracted at 10 µm s^−1^ by > 90 µm to fully separate cell and substrate. Cells were then incubated for 10 min on the cantilever to ensure firm binding. Cells of similar size and morphology were attached to cantilevers to minimize possible variations. The cell morphology was monitored throughout the experiment using optical microscopy to ensure that only cells having round morphologies were characterized.

Cell adhesion forces were quantified by approaching single cells to the protein substrate at 5 µm s^−1^ until recording a compression force of 1, 2, 5, or 10 nN. The cantilever was maintained at constant height for contact times of 5, 20, 60, 120, 240, or 360 s. Thereafter, the cantilever‐bound cell was retracted from the substrate at 5 µm s^−1^ for 100 µm to fully separate cell and substrate. After the experimental cycle, cells were allowed to recover from adhesion measurement for the time of contact time before measuring the adhesion force for a different contact time. The order of contact times was randomized for each cell to exclude potential memory effects of the cells on experimental sequences. The printed protein pattern on the substrate was altered after every adhesion force measurement. Adhesion forces of cantilever bound cells were quantified for all contact times unless morphological changes, such as cell spreading or cell division, were detected. Adhesion forces were determined from retraction force–distance curves after drift‐and baseline correction using JPK data analysis software (JPK data processing 7.0). Adhesion force strengthening was determined as the slope of linear fits to all adhesion forces and contact times (PRISM).

### Confocal Microscopy

Substrates patterned with fluorescently labeled proteins (see *Microcontact printing (µCP) of ECM proteins*) were imagined using confocal microscopy (LCI Plan‐Neofluar 63x/1.3 Imm W Korr DIC objective, Zeiss). To detect the contact area between cells and substrate at different compression forces, an AFM‐based CellHesion200 was mounted on an inverted microscope equipped with a confocal module (AxioObserver equipped with an LSM700, both Zeiss). Cells were grown and detached as described in *Single‐cell force spectroscopy (SCFS)* and incubated in DMEM supplemented CellTracker (CellTracker Fluorescent Probes, C2925, Thermo Fisher Scientific, 1:1000) at 37 °C for 30 min. After incubation, cells were washed with PBS to remove the staining solution and attached to ConA‐coated cantilevers as described. Z‐stack confocal microscopy images (LCI Plan‐Neofluar 63x/1.3 Imm W Korr DIC objective, Zeiss) of uncompressed, cantilever‐bound cells were acquired at 512 × 512 pixels with 0.37 µm intervals. Subsequently, cantilever‐bound cells were brought into contact with the protein substrate at 5 µm s^−1^ until a compression force of 1, 2, 5, or 10 nN was recorded and the cantilever height was maintained constant during image acquisition. For timelapse experiments, cells were maintained at the compression force of 10 nN for 360 s. After imaging, the cantilever‐bound cells were detached from the substrate, allowed to recover for 5 min before acquiring another z‐stack for a different compression force. For analyzing the height of the compressed cells, x‐z plane images were created from maximum intensity projection. The height of each compressed cell was quantified as the distance between cantilever and substrate in the center of the cell using Zen Black 3.9 (Zeiss) in the maximum intensity projection image. For analyzing the contact area of the compressed cells, x‐y plane images were created at the cell‐substrate contact plane. And the contact area was quantified as the area of fluorescent glutathione of cells.

To characterize where integrin mediated adhesion sites form on ECM protein patterns, paxillin‐GFP expressing HeLa cells or fibroblasts were attached to ConA‐coated AFM cantilevers and approached to collagen I or fibronectin patterns having different sizes (printed collagen I patterns having areas of ≈42.2, ≈27.7, and ≈2.6 µm^2^; printed fibronectin patterns having areas of ≈30.5, ≈8.4, and ≈2.3 µm^2^; Figures S6 and S7, Supporting Information). The cantilever‐attached HeLa cells or fibroblasts were brought into contact with the ECM protein patterns on which the cells were compressed with 1 or 10 nN compression force for ≈11 min contact time. During compression paxillin‐GFP localization was monitored every 1 min using confocal microscopy (LSM700 with a LCI Plan‐Neofluar 63x/1.3 Imm Korr DIC objective, Zeiss). Laser intensities and gains were optimized for each cell prior to experiments. After every experiment, the cantilever and the cantilever‐bound cell were exchanged.

### PDMS Stamps for µCP

PDMS stamps having diameters of 8, 5, and 2 µm for µCP were fabricated through replication molding using masters. The silicon master was created via photolithography and ion beam etching processes. Initially, the micropillar pattern was transferred to a UV‐sensitive photoresist by photolithography using a glass mask with the desired pattern. Following the development of the photoresist layer, the silicon substrate was etched to 5 µm depth using ion beam etching. Finally, the photoresist was removed using a plasma asher. Poly(dimethyl) siloxane (PDMS) elastomer (Sylgard 184, Dow corning) was mixed in a 10:1 (w/w) ratio of prepolymer and curing agent and degassed by vacuum for 30 min. The PDMS was casted on ethanol washed silicon masters and cured at 100 °C for 1 h. Peeled‐off PDMS pillar stamps were sonicated in 70% ethanol followed by deionized water washing and dried with pressurized air.

### Microcontact Printing (µCP) of ECM Proteins

Collagen I (PureCol Type I Collagen, 5005, Advanced BioMatrix) or fibronectin (Fibronectin, Bovine Plasma, 341 631, Sigma–Aldrich) were fluorescently labeled by incubating 50 µg mL^−1^ protein solution (in PBS) with Alexa Fluor 555 (Alexa Fluor 555 NHS Ester, A20009, Thermo Fisher Scientific) in a 1:100 (vol/vol) dilution for 10 min at RT. The PDMS stamps were incubated with 50 µg mL^−1^ Alexa fluor 555‐labeled collagen I or fibronectin for 30 min at RT. Inked PDMS stamps were washed twice with PBS and deionized water, and dried by compressed air for 30 s. Subsequently, the PDMS stamp was placed on glass surfaces of a Petri dish (fluoro dish, FD35‐100, World Precision Instruments) for 5 min at RT to allow complete transfer of the protein. For passivation, the glass surfaces incubated with fluorescein isothiocyanate (FITC) conjugate Bovine Serum Albumin (BSA, A9771‐50MG, Sigma Aldrich) or Alexa Fluor 488 (Alexa Fluor 488 NHS Ester, A20000, Thermo Fisher Scientific) ‐labeled FNIII7‐10ΔRGD (50 µg mL^−1^) at 4 °C overnight. Prior to using the dishes in experiments, they were washed with PBS to remove residual proteins.

### Characterization of ECM Protein Patterns

Printed collagen I or fibronectin patterns were characterized by AFM (NanoWizard II, JPK) imaging in contact mode at RT using triangular cantilevers with nominal spring constants of 0.35 N m^−1^ (SNL‐10, Bruker). Patterned surfaces were first imaged 50 µm × 50 µm for an overview image at 256 × 256 pixels at a line rate of 1.8 Hz. Subsequently, single printed ECM protein patterns were imaged at 256 × 256 pixels. The AFM images were collected from at least five different patterned surfaces with 26 printed patterns in total. Gains and cantilever deflection were adjusted during the scans to acquire best possible images at the lowest possible imaging force applied.

Height profiles of printed patterns were obtained from cross‐sections using the AFM inbuild data processing software (JPK data processing 7.0). To measure the diameter of the patterns, average of the full horizontal and vertical width at half average height was measured for individual patterns. To quantify the area of the patterns, a binary height mask of single patterns was created at the half average height using the AFM data processing software. Binary images were imported to ImageJ, transformed into grey scale images and the area of the patterns was quantified using the analyze particles command.

### Glycosidases Cocktail Preparation

Chondroitinase ABC (ChrABC) enzyme from Proteus vulgaris (CHONDROITINASE‐ABC BSA FREE, C3667‐5UN, Merck), hyaluronidase from bovine testes (HYALURONIDASE TYPE I‐S FROM BOVINE, H3506‐100MG, Merck), heparinase I from Flavobacterium heparin (H2519‐50UN, Merck), neuraminidase from Clostridium perfringens (C. welchii) (NEURAMINIDASE FROM#CLOSTRIDIUM PERFRING&, N2876‐6UN, Merck) and tunicamycin from Streptomyces sp. (T7767‐1MG, Merck) were used for the digestion of glycocalyx of HeLa cells and fibroblasts. Stock solution of ChrABC (5 U mL^−1^) was prepared in 0.01% BSA buffer. Hyaluronidase stock solution (100 mg mL^−1^) was prepared in 2 mm phosphate buffer pH 7 with 77 mm NaCl and 0.01% BSA. Heparinase I (50 U mL^−1^) was prepared in 20 mm Tris‐HCl pH 7.5 with 50 mm NaCl and 4 mm CaCl_2_. Neuraminidase (6 U mL^−1^) was prepared in 50 mm Trizma HCl pH 8.0. tunicamycin (10 mg mL^−1^) was prepared in DMSO and stored at 4 °C. All solutions except tunicamycin were stored at −20 °C for less than one month. The used glycosidases cocktail is contained 100 mU mL^−1^ of ChrABC and neuraminidase, 10 mU mL^−1^ of heparinase I, 500 U mL^−1^ hyaluronidase, and 1 µg mL^−1^ of tunicamycin. The cocktail with 20 µL ChrABC, 16.7 µL neuraminidase, 0.2 µL heparinase I, 5 µL hyaluronidase, and 0.1 µL tunicamycin of stock solution was freshly prepared in per mL cell culture medium before use.

### Cells Treating by Glycosidases Cocktail

HeLa cells and fibroblasts were serum starved for at least 1 h prior to experiments. Then cells were washed with PBS and detached with 0.25% (wt/vol) trypsin/EDTA (25 200 072, Thermo Fisher Scientific) for 2 min at 37 °C. Detached cells were suspended in cell culture medium with glycosidases cocktail for 3 h at 37 °C. Control cells were maintained in same condition in the absence of the glycosidase cocktail. After treatment cells were pelleted by centrifugation and were re‐suspended in 200 µl SCFS media prepared by DMEM (powder, high glucose, pyruvate, 12 800 017, Thermo Fisher Scientific) supplemented with 20 mm HEPES with cocktail added.

### Flow Cytometry Test of Glycocalyx Removal Cells

As a negative control, not labeled HeLa cells and fibroblasts were serum starved for at least 1 h, trypsinized, washed with PBS and 6 × 10^5^ cells were resuspended in 500 µL PBS. For characterizing the efficiency of glycosidase cocktail, 1 h glycosidase cocktail‐treated, 3 h glycosidase cocktail‐treated or positive control cells were stained for the glycocalyx by incubating the suspended cells in cell culture medium supplemented with 1 µg mL^−1^ wheat germ agglutinin (Oregon Green 488 conjugate, W6748, Invitrogen) for 30 min at 37 °C and 5% CO_2_. For addressing the role of glycocalyx in activating integrin, HeLa cells and fibroblasts were stained with FITC‐integrin β1 (1:40; anti‐integrin beta 1, 11‐0291‐82, Thermo Fisher Scientific) or rat anti‐9EG7 (553 715, BD Biosciences) followed with donkey anti‐rat Alexa fluor 555 (A48270, Thermo Fisher Scientific). Afterward, cells were pelleted and resuspended in 500 µL PBS. Flow cytometry was carried out using the BD LSR Fortessa SORP (BD Biosciences) with laser 488 nm detecting green fluorescence. Laser intensities were optimized for each experiment and maintained constant for conditions that were compared. Flow cytometry data was gated according to forward and side scatter to exclude debris and doublets. FlowJo V10 was used to analyze the flow cytometry data.

### Statistical Analysis

All adhesion forces were preprocessing as described in *Single‐cell force spectroscopy (SCFS)*. The quantitative results are presented as detailed in the figure legends. Briefly, the sample size *n* (cells) representing the number of independent cells analyzed is specified in each figure legend and encompasses data from at least three independent experiments. Statistical tests, such as indicated in the figure legends, were performed using GraphPad Prism and two‐tailed unpaired, nonparametric Mann‐Whitney tests. To statistically compare adhesion strengthening under different conditions, a linear regression analysis of the adhesion forces recorded for all contact times was performed. The extra sum‐of‐squares *F* test was used to statistically test the adhesion strengthening under different conditions. *P* <0.05 was considered statistically significant.

## Conflict of Interest

The authors declare no conflict of interest.

## Author Contributions

X.W., N.S., D.J.M. designed the experiments and wrote the paper. X.W. performed and analyzed most experiments. J. H. and N.S. helped with experiments set up and data analysis. All authors discussed the experiments, read and approved the manuscript.

## Supporting information



Supporting Information

## Data Availability

The data generated in this study has been depositied in the ETH research data collection and is available under https://hdl.handle.net/20.500.11850/735465.
