# Supplementary material for: Compressed Cells Facilitate Adhesion Through Glycocalyx
Source: Adv Sci (Weinh). 2025 Aug 15;12(35):e13586. doi: 10.1002/advs.202413586 (PMC12462985; doi:10.1002/advs.202413586)
Supplement: Supplementary file 1 — Supporting Information [file ADVS-12-e13586-s001.docx]

Supporting Information

Compressed cells facilitate adhesion through glycocalyx

Xiaole Wang, Jonne Helenius, Daniel J. Müller*, Nico Strohmeyer*


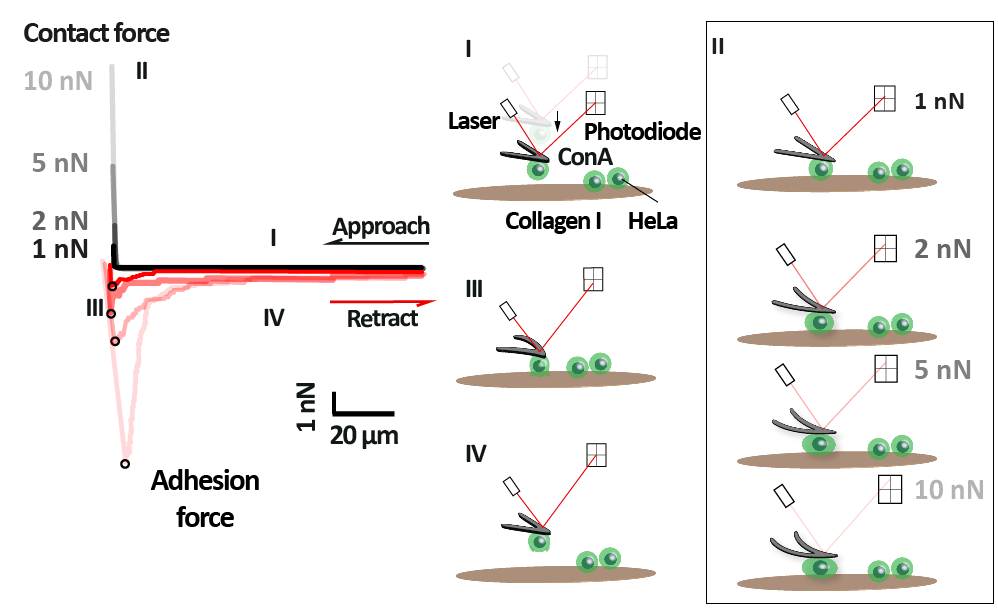


**Figure S1.** **AFM-based single-cell force spectroscopy (SCFS) setup to measure contact area and compression force dependent cell adhesion forces to ECM substrates.** (I-V) SCFS measures the adhesion force of a single living cell to a substrate of interest. First, a ConA-coated microcantilever is gently pushed onto a suspended cell to attach it to the microcantilever. After 5 s, the cell attached to the microcantilever and is withdrawn from the substrate. Thereafter, the microcantilever-bound cell is positioned above the substrate (I) and approached to the substrate until reaching a contact or compression force of 1 nN, 2 nN, 5 nN and 10 nN (II). At this position, the microcantilever is maintained at a constant height for the contact time ranging from 5 to 360 s. Subsequently, the microcantilever is retracted (III) to detach the cell from the substrate (IV). During approaching to and retracting from the substrate, the deflection of the microcantilever is recorded through a laser, which is reflected from the back of the microcantilever into a position sensitive photodiode. The force deflecting the microcantilever and the distance travelled by the microcantilever is recorded in force-distance (FD) curves. The maximum downward deflection in the retraction FD curve quantifies the maximum adhesion force of the cell to the substrate.^[1]^


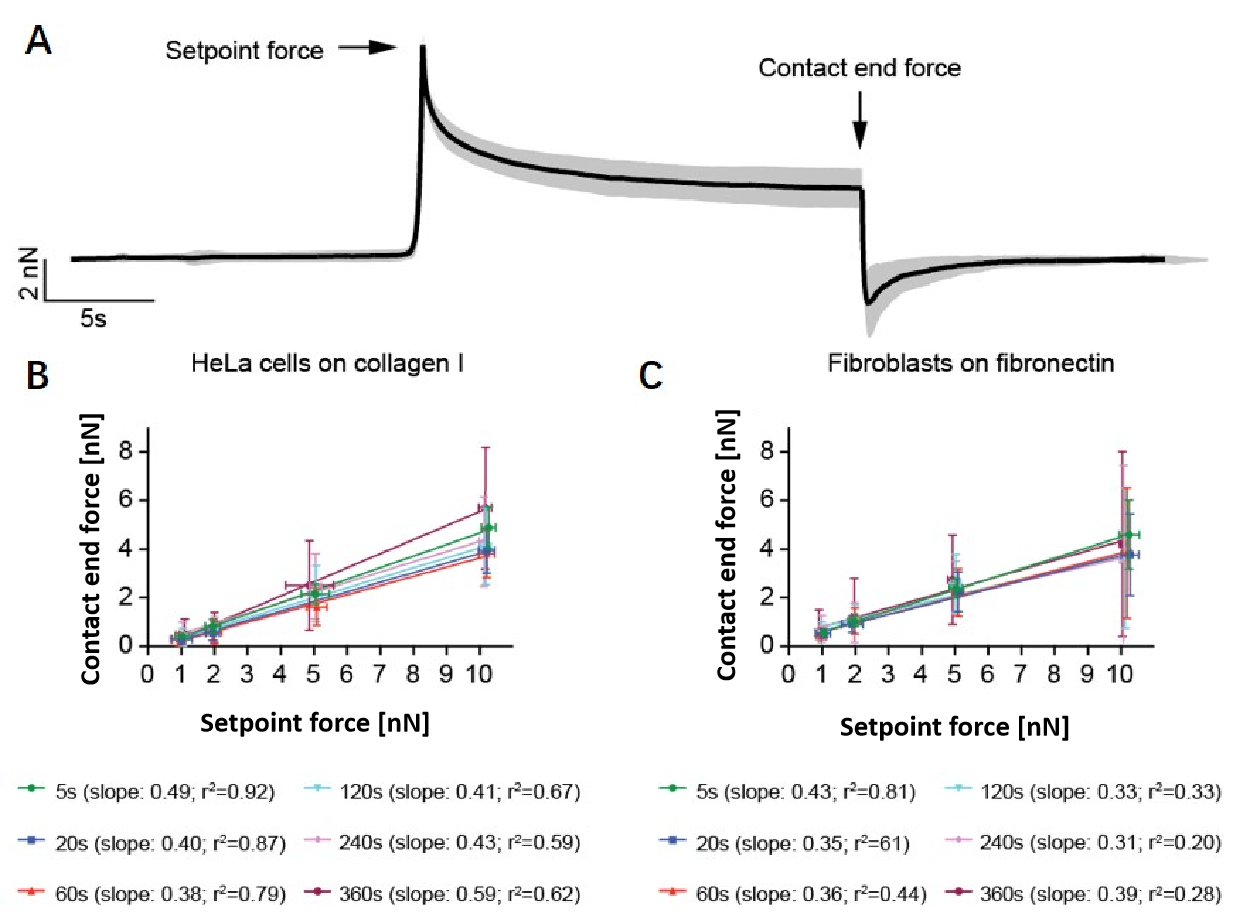


**Figure S2. Contact end force linearly scales with the setpoint force (compression force) in SCFS experiments.** (A) Representative average force-time curves (black lines) with SD (grey area) of SCFS experiments compressing single HeLa cells (*n* = 20) onto collagen I-coated supports with 10 nN setpoint force (compression force). (B,C) Correlation of setpoint force and contact end force of (B) HeLa cells to collagen I and (C) fibroblasts to fibronectin (*n* ≥ 13 cells per condition). Dots represent the mean contact end force at the given setpoint force, error bars depict SD, and lines are linear fits for each contact time. The slope is given in the legend and r^2^ represents the goodness of the fit.


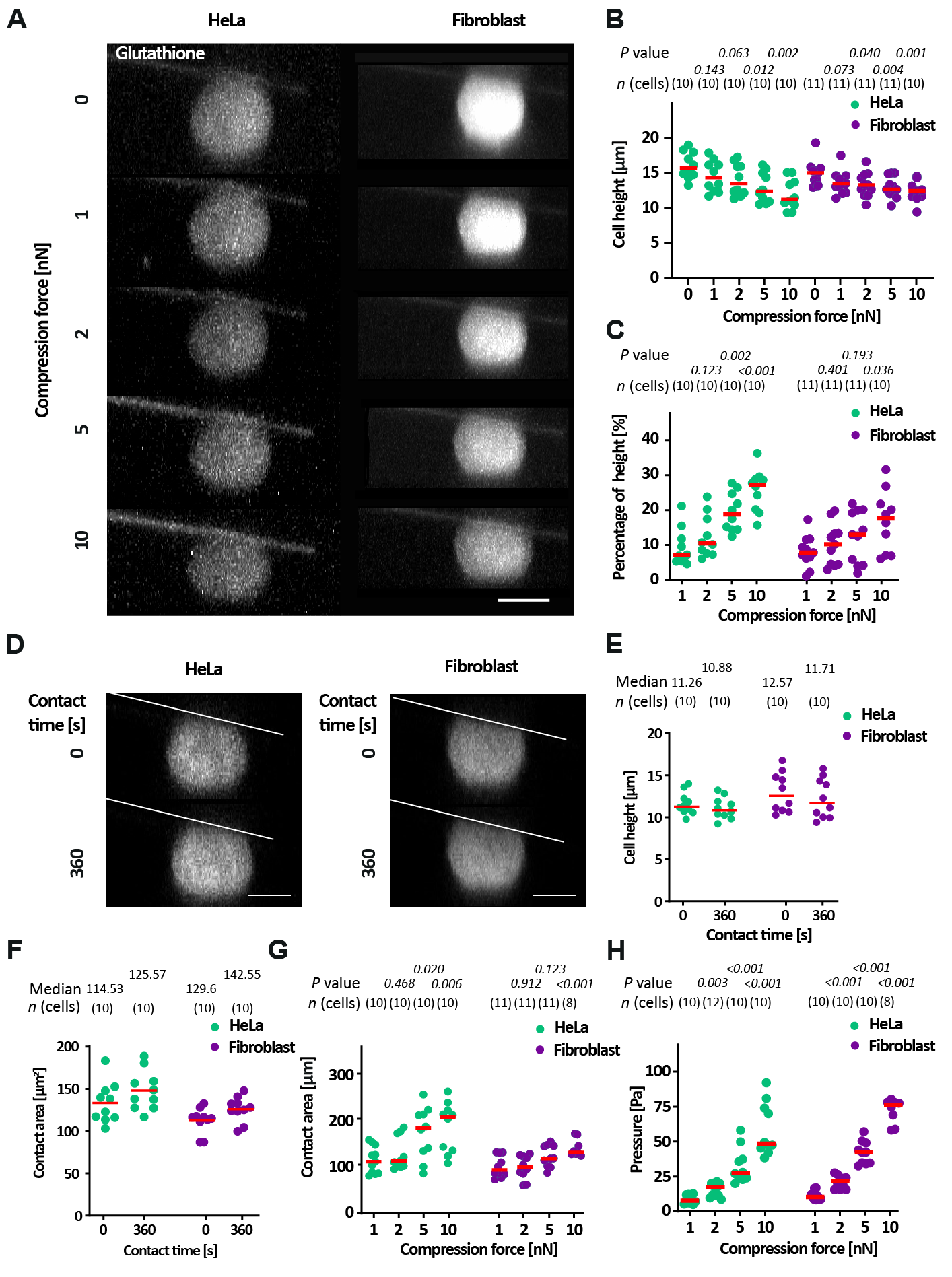


Figure S3. **Characterization of the height of HeLa cells and fibroblasts compressed by different forces with a microcantilever.** (A) Maximum intensity x-z-projection of glutathione labeled (CellTracker) HeLa cells and fibroblasts under different compression forces applied in SCFS. Scale bar, 10 μm. (B-C) Statistical analysis of (B) the height of the compressed cell, (C) the percentage by which the cell height is compressed of HeLa cells (green) and fibroblasts (purple) exposed to different compression forces. Dots represent measurements of single cells, red bars median values, and *n* (cells) the number of independent cells tested in at least three independent experiments. *P* values were calculated by two-sided Mann-Whitney tests and compare the values with those measured at (B) 0 nN compression force or (C) 1 nN compression force. (D) Maximum intensity x-z-projection of glutathione labeled (CellTracker) HeLa cells and fibroblasts at indicated contact times under 10 nN compression force applied in SCFS. The white line depicts the compressing cantilever. Scale bars, 10 μm, (E-F) Statistical analysis of (E) cell height and contact area quantified from (D) confocal z-stack images of HeLa cells (green) and fibroblasts (purple). (G,H) Statistical analysis of (G) the contact area between cell and substrate and (H) the externally applied pressure quantified from (A) confocal z-stack images of HeLa cells (green) and fibroblasts (purple). (E-H) Values were quantified from confocal images and compression forces. Dots represent measurements of single cells, red bars median values, and *n* (cells) the number of independent cells tested in at least three independent experiments. (G,H) *P* values were calculated by two-sided Mann-Whitney tests and compare the values with those measured at 1 nN compression force.


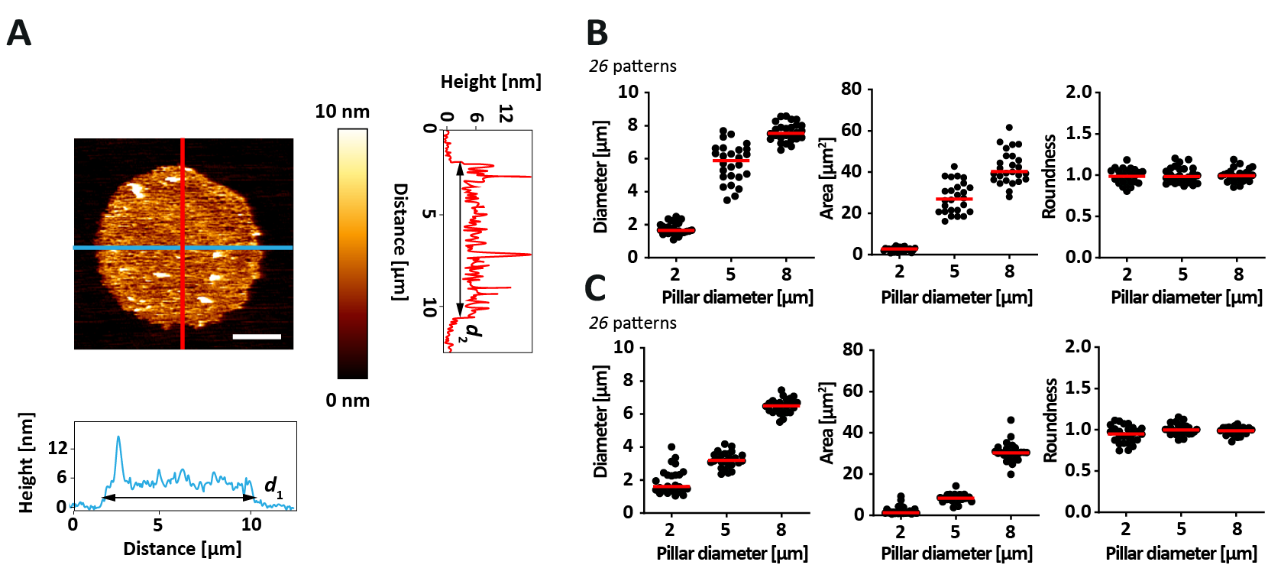


Figure S4. **Characterization of printed ECM protein patterns.** (A) Representative AFM height image (topograph) of a fibronectin pattern printed on glass using a PDMS pillar having a diameter of 8 µm. Perpendicular diameters (*d*_1_, *d*_2_) of the printed ECM protein patterns were used to quantify the average diameter, area, and roundness of the printed patterns. The topograph was recorded using contact mode AFM in buffer solution (PBS) and upon applying an imaging force of 0.67 nN. Scale bar, 2.5 µm. (B) Diameter, area, and roundness derived from 26 (A) collagen I or (B) fibronectin patterns, which were printed using PDMS micropillars of given diameters. Dots represent values of individual printed patterns, red bar mean values.


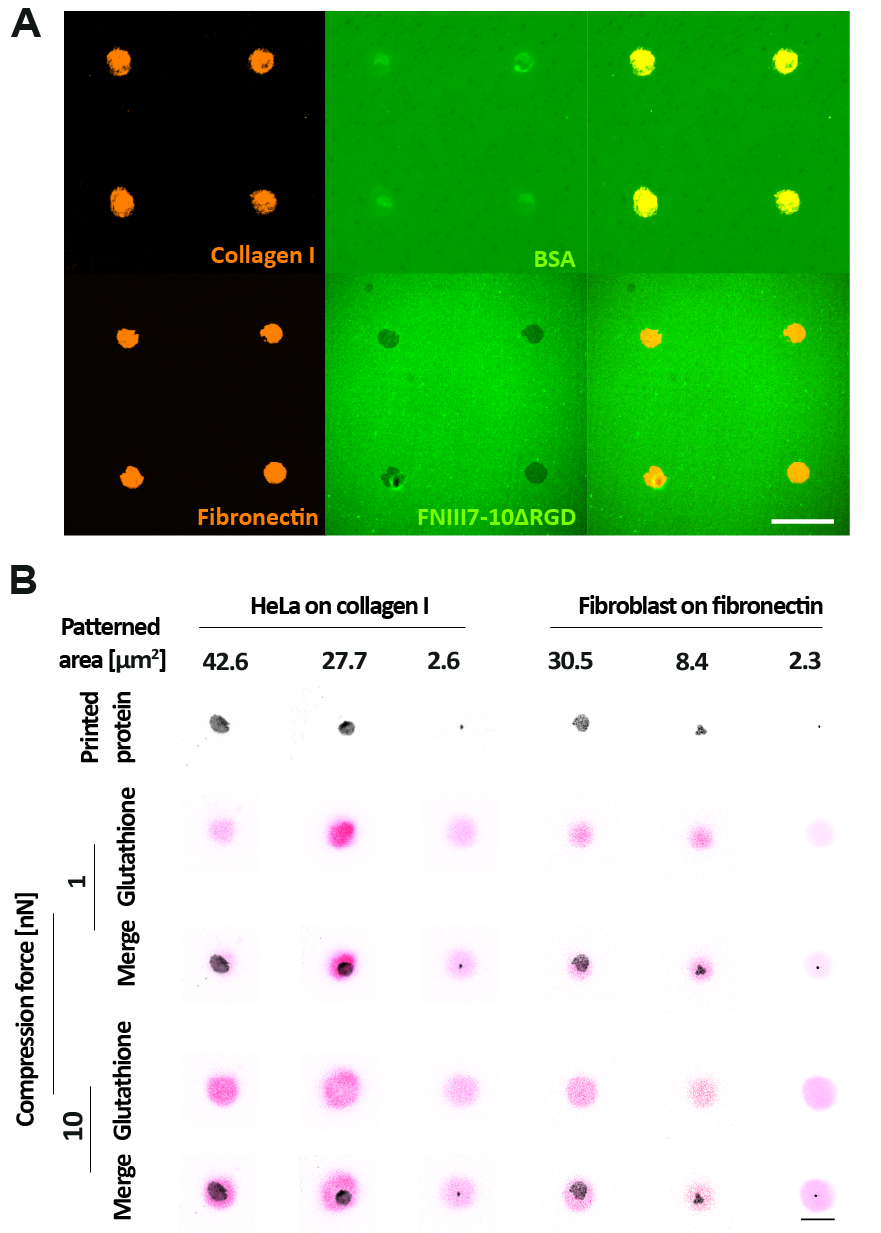


Figure S5. **Contact area of HeLa cells and fibroblasts compressed to printed ECM protein substrates.** (A) Confocal microscopy images of printed Alexa fluor 555-labeled collagen (top, left) or fibronectin (bottom, left) patterns passivated with FITC-labelled BSA (top, middle) or Alexa fluor 488-labelled FNIII7-10ΔRGD (bottom, middle). The right images show superimpositions of left and middle fluorescence microscopy images. *n* = 3 independent experiments. Scale bar, 20 μm. (B) Representative confocal microscopy images of the contact area of a microcantilever-bound (left) HeLa cell or (right) fibroblast to different printed ECM pattern areas at given compression force. Glutathione of cells were fluorescently labelled by CellTracker (pink) and printed collagen I or fibronectin patterns by Alexa fluor 555 (gray). *n* = 3 independent experiments. Scale bar, 12 μm.


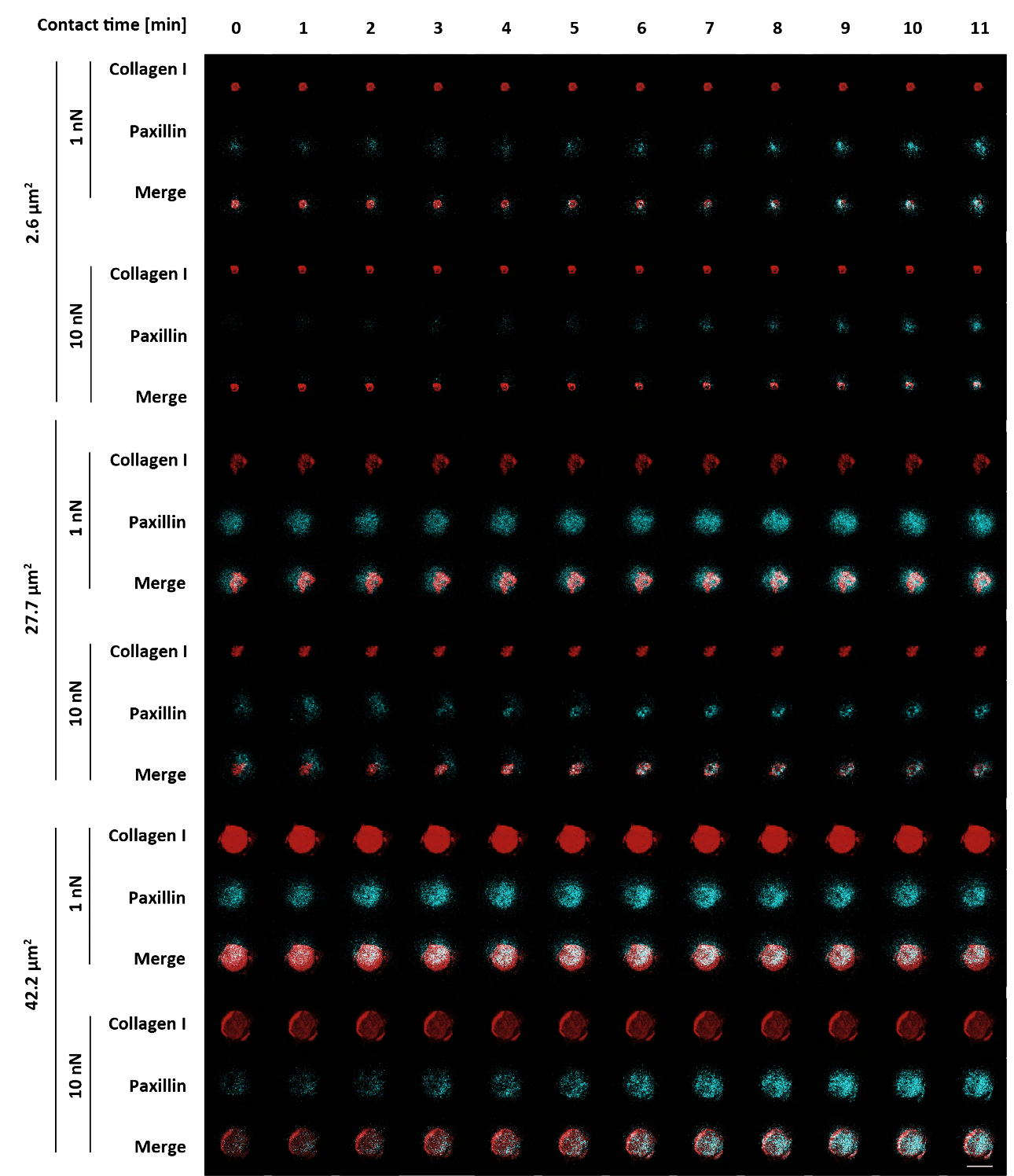


**Figure S6**. **The formation of adhesion sites by HeLa cells on collagen I patterns is independent of the compression force.** Timelapse confocal microscopy images of paxillin-GFP expressing HeLa cells compressed on collagen I patterns. Pattern area, compression force, and contact time are indicated. A single, rounded paxillin-GFP expressing HeLa cell was attached to a ConA-coated AFM cantilever and then brought into contact with collagen I patterns until reaching contact times of ≈ 11 min. The force at which the single cells were compressed on the pattern was either 1 or 10 nN. Collagen I patterns were labelled using Alexa fluor 555 (gray). *n* = 3 independent experiments. Scale bars, 7 μm.


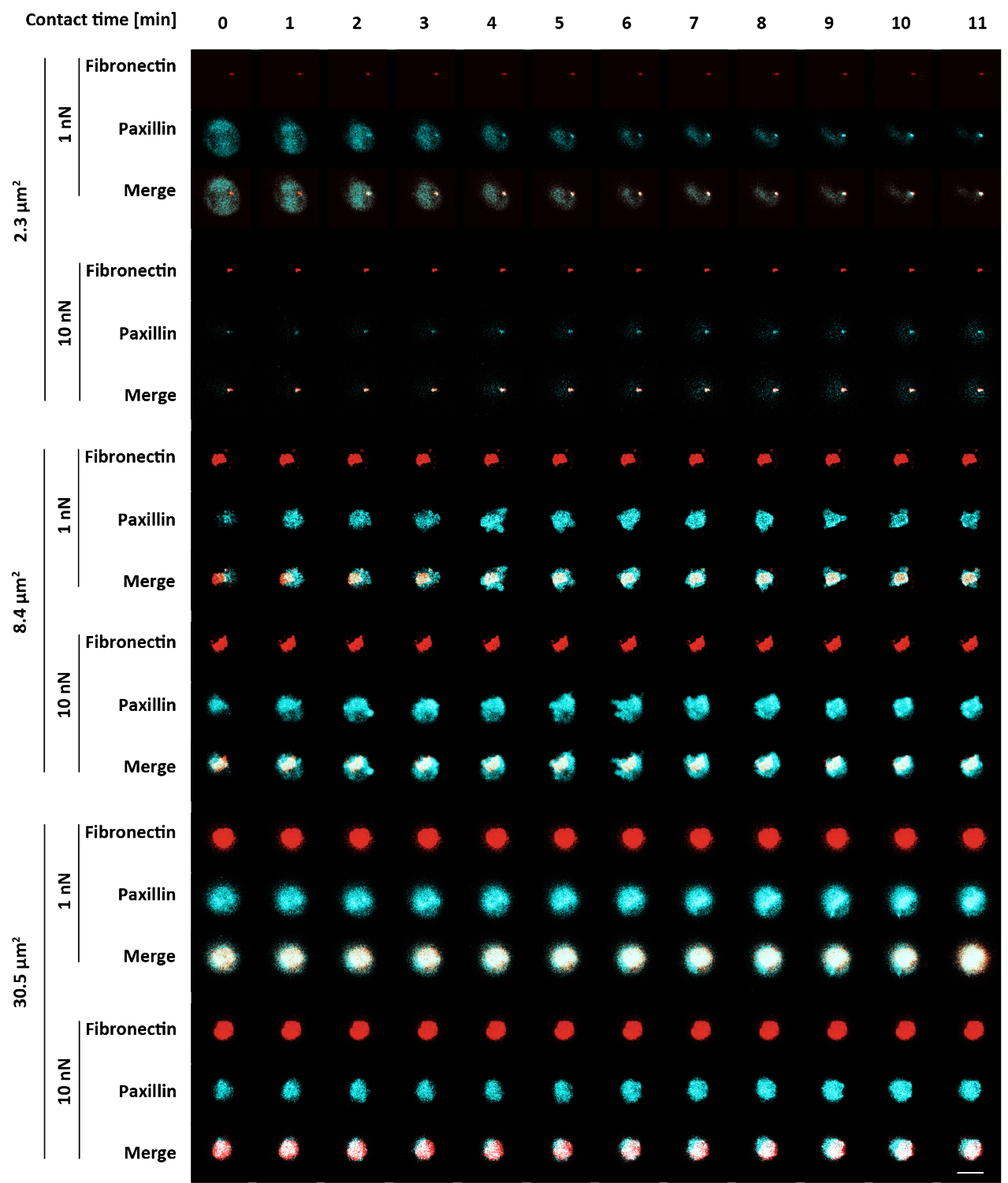


**Figure S7**. **The formation of adhesion sites by fibroblasts on fibronectin patterns is independent of the compression force.** Timelapse confocal microscopy images of paxillin-GFP expressing fibroblasts compressed on fibronectin patterns. Pattern areas, compression forces and contact times are indicated. A single, rounded paxillin-GFP expressing fibroblast was attached to a ConA-coated AFM cantilever and then brought into contact with fibronectin patterns until reaching contact times of ≈ 11 min. The force at which the single cells were compressed on the pattern was either 1 or 10 nN. Fibronectin patterns were labelled using Alexa fluor 555 (gray). *n* = 3 independent experiments. Scale bars, 7 μm.


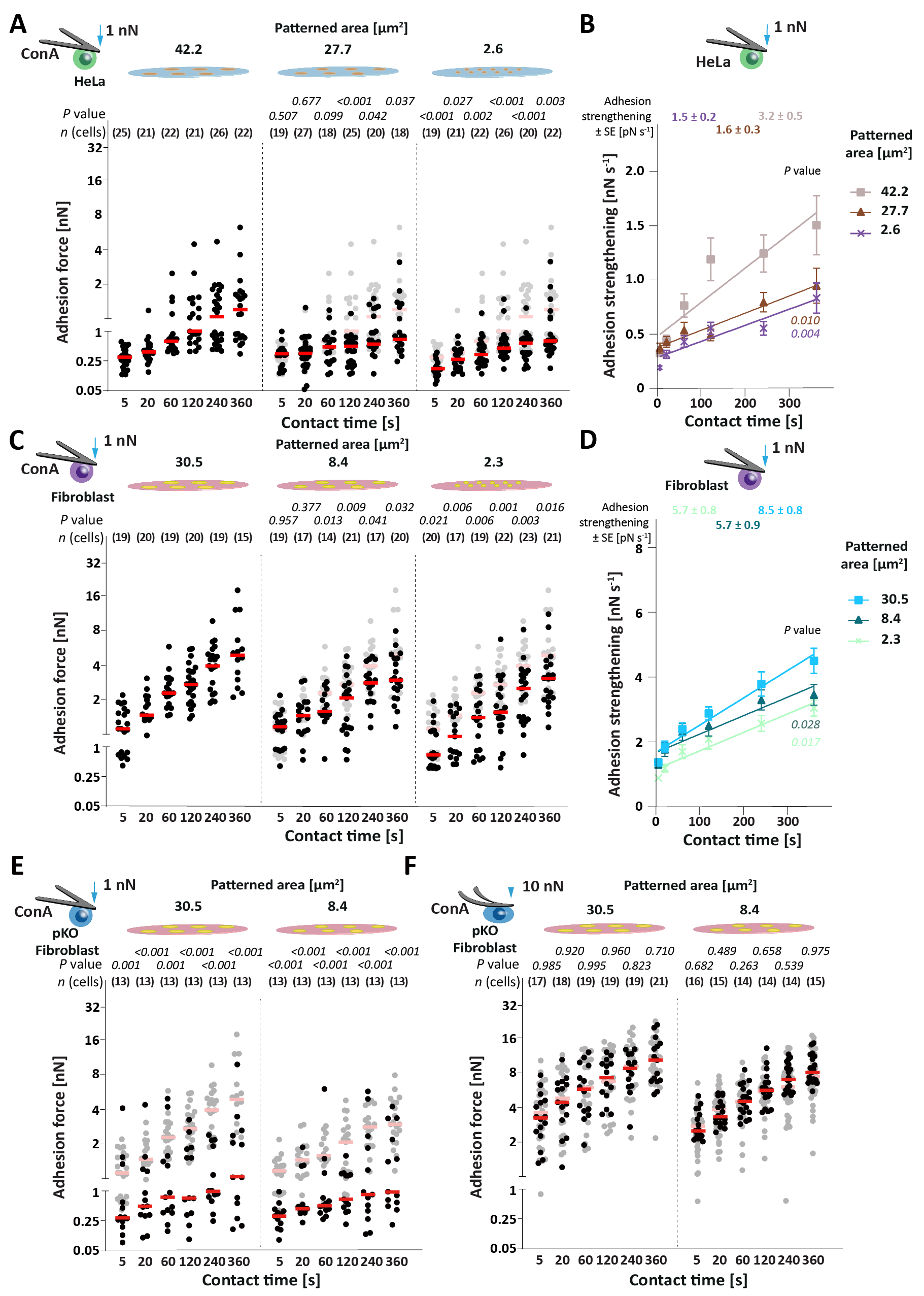


Figure S8. Cell adhesion force depends on ECM pattern area. (A,C) Adhesion force of (A) HeLa cells and of (C) fibroblasts to printed collagen I or fibronectin patterns under 1 nN compression force. Dots represent adhesion forces of single cells, red bars median values, and *n* (cells) the number of independent cells tested in at least three independent experiments. Adhesion force of (A) HeLa cells to printed collagen I patterns having areas of 42.2 μm^2^ or (C) fibroblasts to printed fibronectin patterns having areas of 30.5 μm^2^ are given as reference (semitransparent). *P* values were calculated by two-sided Mann-Whitney tests and compare the adhesion force of displayed and reference data. (B,D) Adhesion strengthening rate of (B) HeLa cells and of (D) fibroblasts as quantified by the slope of a linear regression fit of the adhesion force (data taken from A and C). Numbers give the slope and standard error (SE) of the linear fit that describe the adhesion strengthening rate. Dots depict mean adhesion forces, error bars SEM, and lines linear regressions. *P* values were calculated by extra sum squares *F* test and compare the slopes at the indicated compression force with the slope of (B) HeLa cells to printed collagen I patterns having areas of 42.2 μm^2^ or of (D) fibroblasts to printed fibronectin patterns having areas of 30.5 μm^2^. (E,F) Adhesion force of pKO fibroblasts to printed fibronectin patterns of given sizes under (E) low (1 nN) and (F) high (10 nN) compression force. Adhesion force under low and high compression force of wild-type fibroblasts to fibronectin patterns having indicated areas is given as reference (semitransparent; data taken from Figure 3C). Dots represent adhesion forces of single cells, red bars median values, and *n*(cells) the number of independent cells tested in at least three independent experiments. *P* values comparing the adhesion force of pKO fibroblasts on indicated fibronectin patterns with reference data were calculated by two sided Mann-Whitney tests.


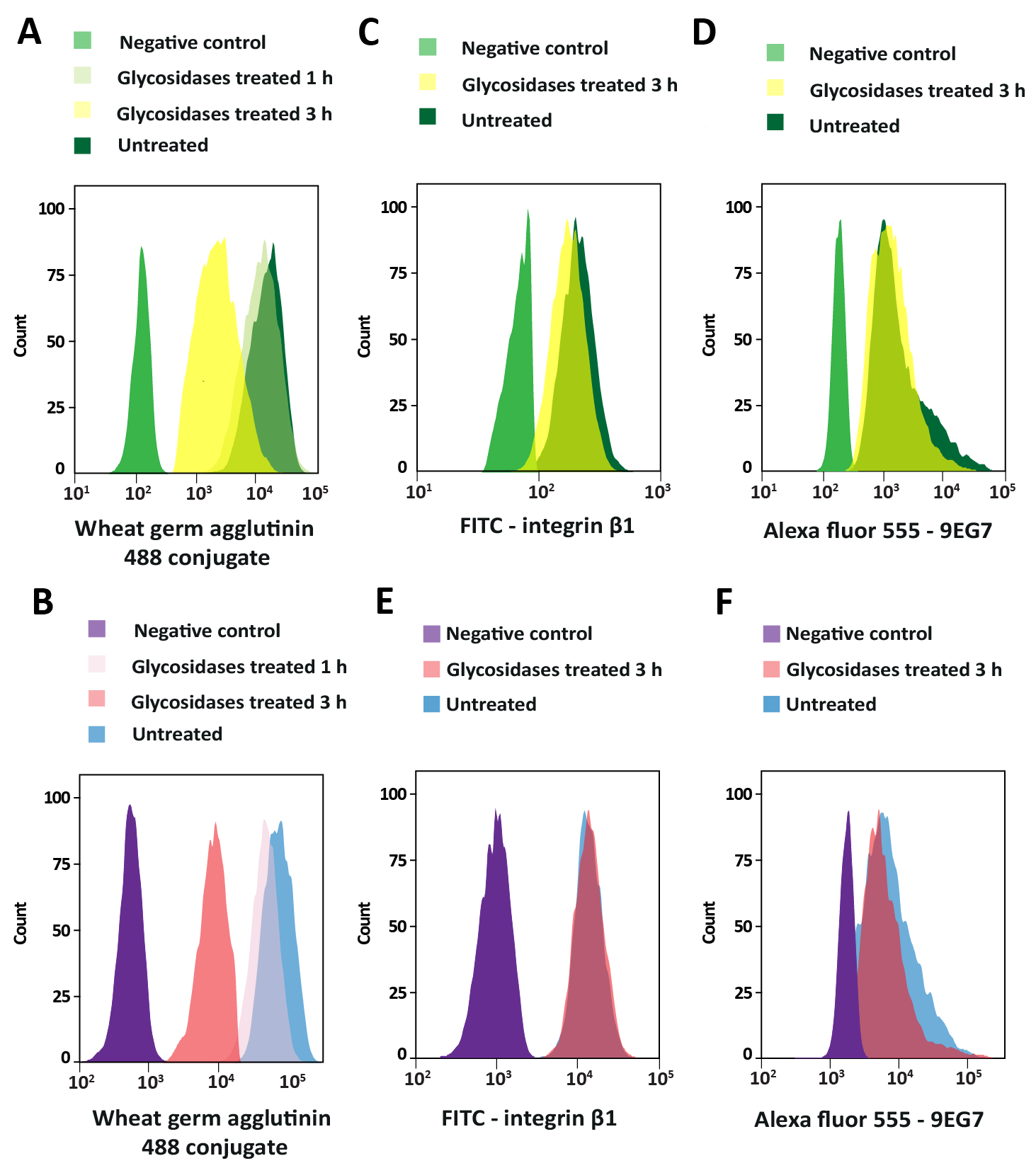


Figure S9. Glycocalyx removal confirmed by flowcytometry. (A,B) Fluorescence intensity of (A) HeLa cells or (B) fibroblasts incubated with glycosidase-cocktail for 1 h or 3 h. Cells were stained by Oregon green 488-conjugated wheat germ agglutinin. As negative controls unstained (A) HeLa cells and (B) fibroblasts were used. (C-F) Fluorescence intensity of glycosidase-cocktail treated (3 h) and untreated (C,D) HeLa cells or (E,F) fibroblasts stained with (C,E) FITC-integrin β1 or (D,F) rat anti-9EG7 followed with donkey anti-rat Alexa fluor 555. As negative controls unstained (C,D) HeLa cells and (E,F) fibroblasts were used. *n* = 3 independent experiments.


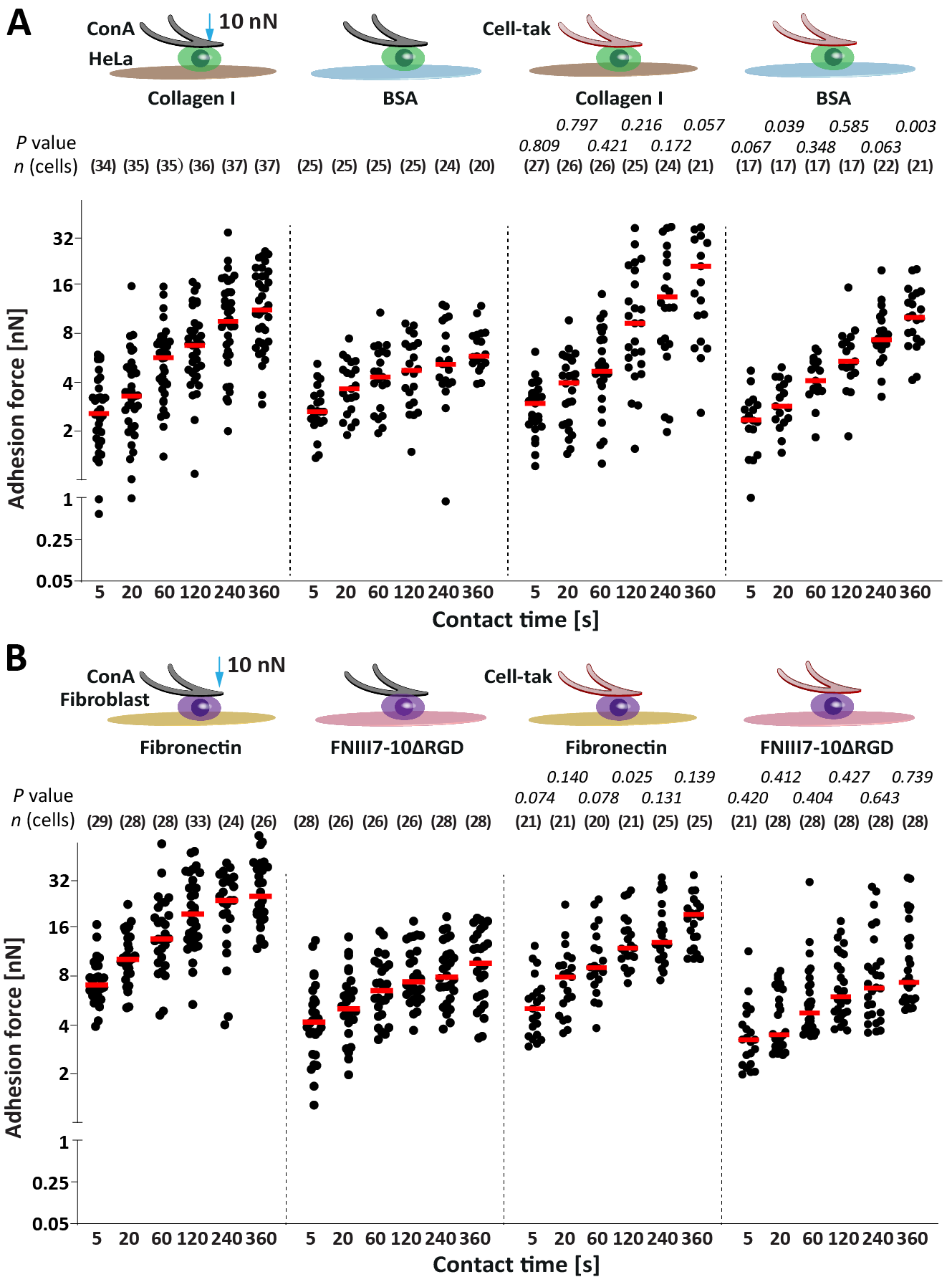


Figure S10. Cell attachment to microcantilever does not affect cell adhesion. Adhesion force of (A) HeLa cells attached to ConA-coated (left) or Cell-tak-coated (right) microcantilever to collagen I or BSA substrates and of (B) fibroblasts attached to ConA-coated (left) or Cell-tak-coated (right) microcantilever to fibronectin or FNIII7-10ΔRGD substrates under 10 nN compression force. Dots represent adhesion forces of single cells, red bars median values, and *n* (cells) the number of independent cells tested in at least three independent experiments. *P* values were calculated by two-sided Mann-Whitney tests and compare the adhesion force of cells attached to Cell-tak and ConA-coated microcantilevers. Data taken from Figure 1A-D and Figure 4A-D.


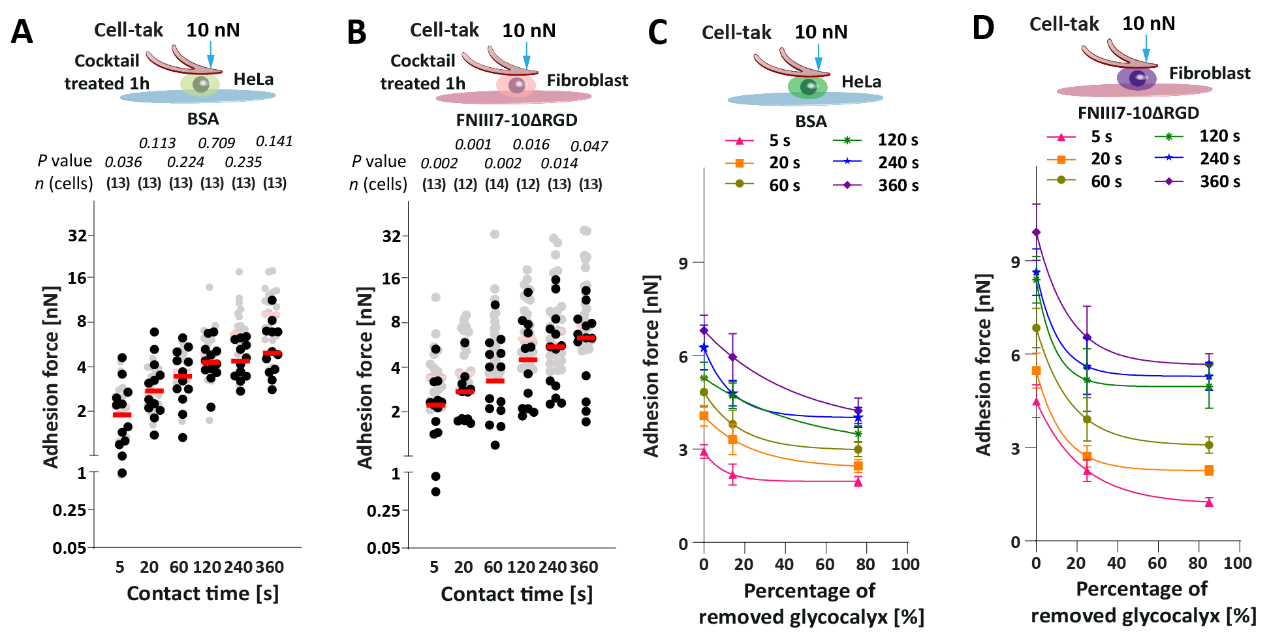


Figure S11. At high compression, the unspecific adhesion force exponentially decays with the percentage of removed glycocalyx. (A,B) Adhesion force of 1 h glycosidase-cocktail treated (A) HeLa cells to BSA or (B) fibroblasts to FNIII7-10ΔRGD at 10 nN compression force (high compression). Dots represent adhesion forces of single cells, red bars median values, and *n* (cells) the number of independent cells tested in at least three independent experiments. *P* values were calculated using two sided Mann-Whitney tests and compare the adhesion force of the given data set with that of untreated (A) HeLa cells to BSA and (B) fibroblasts to FNIII7-10ΔRGD at 10 nN compression force (semitransparent). Data taken from Figure 4C,D. (C,D) Dependence of adhesion force of (C) HeLa cells to BSA and (D) fibroblasts to FNIII7-10ΔRGD at 10 nN compression force on the removal of glycocalyx at different contact times. Dots depict mean adhesion force, error bars SEM. Lines show one phase decay fits of the adhesion force measured of untreated, 1 h and 3 h glycosidase-cocktail treated cells.

| **Comparison of the adhesion force of HeLa cells to collagen I or BSA substrates** | | | | | | | | | |  |
| --- | --- | --- | --- | --- | --- | --- | --- | --- | --- | --- |
| **Collagen I substrate** | | | | | | | |  | |  |
| **Compression** **force [nN]** |  | **1** | |  |  |  |  | |  | |
| Contact time [s] |  | 5 | | 20 | 60 | 120 | 240 | | 360 | |
| Compression force 2 nN | *P* value | 0.044 | | 0.069 | 0.259 | 0.651 | 0.485 | | 0.141 | |
| Compression force 5 nN | *P* value | <0.001 | | <0.001 | <0.001 | <0.001 | <0.001 | | <0.001 | |
| Compression force 10 nN | *P* value | <0.001 | | <0.001 | <0.001 | <0.001 | <0.001 | | <0.001 | |
| **Compression** **force [nN]** |  | **2** | |  |  |  |  | |  | |
| Contact time [s] |  | 5 | | 20 | 60 | 120 | 240 | | 360 | |
| Compression force 5 nN | *P* value | <0.001 | | 0.003 | 0.002 | 0.007 | 0.008 | | 0.005 | |
| Compression force 10 nN | *P* value | <0.001 | | <0.001 | <0.001 | <0.001 | <0.001 | | <0.001 | |
| **Compression** **force [nN]** |  | **5** | |  |  |  |  | |  | |
| Contact time [s] |  | 5 | | 20 | 60 | 120 | 240 | | 360 | |
| Compression force 10 nN | *P* value | <0.001 | | <0.001 | <0.001 | <0.001 | <0.001 | | <0.001 | |
| **BSA substrate** | | |  | | | | | | |  |
| **Compression** **force [nN]** |  | **1** | |  |  |  |  | |  | |
| Contact time [s] |  | 5 | | 20 | 60 | 120 | 240 | | 360 | |
| Compression force 2 nN | *P* value | <0.001 | | 0.001 | 0.001 | <0.001 | 0.115 | | 0.001 | |
| Compression force 5 nN | *P* value | <0.001 | | <0.001 | <0.001 | <0.001 | 0.002 | | <0.001 | |
| Compression force 10 nN | *P* value | <0.001 | | <0.001 | <0.001 | <0.001 | <0.001 | | <0.001 | |
| **Compression** **force [nN]** |  | **2** | |  |  |  |  | |  | |
| Contact time [s] |  | 5 | | 20 | 60 | 120 | 240 | | 360 | |
| Compression force 5 nN | *P* value | <0.001 | | 0.001 | 0.001 | <0.001 | 0.044 | | 0.016 | |
| Compression force 10 nN | *P* value | <0.001 | | <0.001 | <0.001 | <0.001 | <0.001 | | <0.001 | |
| **Compression** **force [nN]** |  | **5** | |  |  |  |  | |  | |
| Contact time [s] |  | 5 | | 20 | 60 | 120 | 240 | | 360 | |
| Compression force 10 nN | *P* value | <0.001 | | <0.001 | <0.001 | <0.001 | <0.001 | | <0.001 | |

Table S1. **Statistical analysis comparing the adhesion force of HeLa cells to collagen I or BSA substrates with indicated compression force and contact time**. *P* values compare adhesion force of HeLa cells to collagen I or BSA substrate with that measured under the indicated compression force for a given contact time. *P* values were calculated using two-sided Mann-Whitney tests, black values show significant differences (*P* < 0.05), red values non-significant differences (*P* ≥ 0.05). Data taken from Figure 1A,C.

| **Comparison of the adhesion force of fibroblasts to fibronectin or FNIII7-10ΔRGD substrates** | | | | | | | | | |
| --- | --- | --- | --- | --- | --- | --- | --- | --- | --- |
| **Fibronectin substrate** | | | | | | | | |  |
| **Compression** **force [nN]** |  | **1** | |  |  |  |  |  | |
| Contact time [s] |  | 5 | | 20 | 60 | 120 | 240 | 360 | |
| Compression force 2 nN | *P* value | 0.093 | | 0.104 | 0.078 | 0.564 | 0.324 | 0.09 | |
| Compression force 5 nN | *P* value | <0.001 | | <0.001 | <0.001 | <0.001 | <0.001 | <0.001 | |
| Compression force 10 nN | *P* value | <0.001 | | <0.001 | <0.001 | <0.001 | <0.001 | <0.001 | |
| **Compression** **force [nN]** |  | **2** | |  |  |  |  |  | |
| Contact time [s] |  | 5 | | 20 | 60 | 120 | 240 | 360 | |
| Compression force 5 nN | *P* value | <0.001 | | <0.001 | <0.001 | <0.001 | <0.001 | <0.001 | |
| Compression force 10 nN | *P* value | <0.001 | | <0.001 | <0.001 | <0.001 | <0.001 | <0.001 | |
| **Compression** **force [nN]** |  | **5** | |  |  |  |  |  | |
| Contact time [s] |  | 5 | | 20 | 60 | 120 | 240 | 360 | |
| Compression force 10 nN | *P* value | <0.001 | | <0.001 | <0.001 | <0.001 | <0.001 | <0.001 | |
| **FNIII7-10ΔRGD substrate** | | |  | | | | | | |
| **Compression** **force [nN]** |  | **1** | |  |  |  |  |  | |
| Contact time [s] |  | 5 | | 20 | 60 | 120 | 240 | 360 | |
| Compression force 2 nN | *P* value | 0.208 | | 0.381 | 0.073 | 0.003 | 0.127 | 0.335 | |
| Compression force 5 nN | *P* value | <0.001 | | <0.001 | <0.001 | <0.001 | <0.001 | 0.006 | |
| Compression force 10 nN | *P* value | <0.001 | | <0.001 | <0.001 | <0.001 | <0.001 | <0.001 | |
| **Compression** **force [nN]** |  | **2** | |  |  |  |  |  | |
| Contact time [s] |  | 5 | | 20 | 60 | 120 | 240 | 360 | |
| Compression force 5 nN | *P* value | <0.001 | | <0.001 | 0.003 | 0.007 | 0.028 | 0.004 | |
| Compression force 10 nN | *P* value | <0.001 | | <0.001 | <0.001 | <0.001 | <0.001 | <0.001 | |
| **Compression** **force [nN]** |  | **5** | |  |  |  |  |  | |
| Contact time [s] |  | 5 | | 20 | 60 | 120 | 240 | 360 | |
| Compression force 10 nN | *P* value | <0.001 | | <0.001 | <0.001 | <0.001 | <0.001 | <0.001 | |

Table S2. **Statistical analysis comparing the adhesion force of fibroblasts to fibronectin or FNIII7-10ΔRGD substrates at indicated compression force and contact time**. *P* values compare adhesion force of fibroblasts to fibronectin or FNIII7-10ΔRGD substrates under a given compression force with that measured under the indicated compression force for a given contact time. *P* values were calculated using two-sided Mann-Whitney tests, black values show significant difference (*P* < 0.05), red values non-significant difference (*P* ≥ 0.05). Data taken from Figure 1B,D.

| **Comparison of the adhesion strengthening rate of HeLa cells** | | | | |
| --- | --- | --- | --- | --- |
| **Collagen I substrate** | | | | |
| Compression force | | 2 nN | 5 nN |  |
| 5 nN | *P* value | 0.038 |  |  |
| 10 nN | *P* value | <0.001 | <0.001 |  |
| **BSA substrate** | | | | |
| Compression force | | 2 nN | 5 nN |  |
| 5 nN | *P* value | 0.369 |  |  |
| 10 nN | *P* value | <0.001 | <0.001 |  |
| **Comparison of the adhesion strengthening rate of fibroblasts** | | | | |
| **Fibronectin substrate** | | | | |
| Compression force | | 2 nN | 5 nN |  |
| 5 nN | *P* value | 0.036 |  |  |
| 10 nN | *P* value | <0.001 | <0.001 |  |
| **FNIII7-10ΔRGD substrate** | | | | |
| Compression force | | 2 nN | 5 nN |  |
| 5 nN | *P* value | 0.671 |  |  |
| 10 nN | *P* value | <0.001 | <0.001 |  |

**Table S3. Statistical analysis comparing the adhesion strengthening rate of HeLa cells to collagen I and BSA substrates or of fibroblasts to fibronectin and FNIII7-10ΔRGD substrates.** Adhesion strengthening rates of HeLa cells and fibroblasts were quantified by the slope of a linear regression fit of the adhesion force to protein substrates for all contact times. *P* values compare the adhesion strengthening rates of HeLa cells or fibroblasts to indicated ECM substrates under given compression force. *P* values were calculated using extra sum-of-squares *F* test, black values show significant difference (*P* < 0.05), and red values non-significant difference (*P* ≥ 0.05)*.* Data taken from Figure 1E-H.

| **Comparison of the adhesion force of and EDTA-/ non-treated cells to protein substrates** | | | | | | | | | | | | | | | | |  |
| --- | --- | --- | --- | --- | --- | --- | --- | --- | --- | --- | --- | --- | --- | --- | --- | --- | --- |
| **HeLa cells** | | | | | | | | | | | | | |  | | |  |
| Compression force 1 nN | | | | | | | | | | | | | |  | | |  |
|  |  | | EDTA-treated HeLa cells to collagen I | | | | | | | | | | | | | |  |
| Contact time [s] |  | | 5 | | 20 | | 60 | | 120 | | 240 | | 360 | | | |  |
| HeLa cells to BSA | *P* value | | 0.002 | | 0.002 | | 0.113 | | 0.157 | | 0.394 | | 0.103 | | | |  |
| Compression force 2 nN | | | | | | | | | | | | | |  | | |  |
|  |  | | EDTA-treated HeLa cells to collagen I | | | | | | | | | | | | | |  |
| Contact time [s] |  | | 5 | | 20 | | 60 | | 120 | | 240 | | 360 | | | |  |
| HeLa cells to BSA | *P* value | | 0.386 | | 0.711 | | 0.395 | | 0.707 | | 0.805 | | 0.724 | | | |  |
| Compression force 5 nN | | | | | | | | | | | | | | | |  | |
|  | |  | | EDTA-treated HeLa cells to collagen I | | | | | | | | | | | | | |
| Contact time [s] | |  | | 5 | | 20 | | 60 | | 120 | | 240 | | | 360 | | |
| HeLa cells to BSA | | *P* value | | 0.174 | | 0.769 | | 0.023 | | 0.795 | | 0.727 | | | 0.003 | | |
| Compression force 10 nN | | | | | | | | | | | | | | | |  | |
|  | |  | | EDTA-treated HeLa cells to collagen I | | | | | | | | | | | | | |
| Contact time [s] | |  | | 5 | | 20 | | 60 | | 120 | | 240 | | | 360 | | |
| HeLa cells to BSA | | *P* value | | 0.002 | | 0.011 | | 0.160 | | 0.612 | | 0.518 | | | 0.552 | | |
| **Fibroblasts** | | | | | | | | | | | | | |  | | |  |
| Compression force 1 nN | | | | | | | | | | | | | |  | | |  |
|  |  | | EDTA-treated fibroblasts to fibronectin | | | | | | | | | | | | | |  |
| Contact time [s] |  | | 5 | | 20 | | 60 | | 120 | | 240 | | 360 | | | |  |
| Fibroblasts to FNIII7-10ΔRGD | *P* value | | 0.136 | | 0.824 | | 0.283 | | 0.084 | | 0.928 | | 0.719 | | | |  |
| Compression force 2 nN | | | | | | | | | | | | | |  | | |  |
|  |  | | EDTA-treated fibroblasts to fibronectin | | | | | | | | | | | | | |  |
| Contact time [s] |  | | 5 | | 20 | | 60 | | 120 | | 240 | | 360 | | | |  |
| Fibroblasts to FNIII7-10ΔRGD | *P* value | | 0.868 | | 0.755 | | 0.482 | | 0.644 | | 0.536 | | 0.363 | | | |  |
| Compression force 5 nN | | | | | | | | | | | | | | | |  | |
|  | |  | | EDTA-treated fibroblasts to fibronectin | | | | | | | | | | | | | |
| Contact time [s] | |  | | 5 | | 20 | | 60 | | 120 | | 240 | | | 360 | | |
| Fibroblasts to FNIII7-10ΔRGD | | *P* value | | 0.447 | | 0.415 | | 0.366 | | 0.925 | | 0.612 | | | 0.710 | | |
| Compression force 10 nN | | | | | | | | | | | | | | | |  | |
|  | |  | | EDTA-treated fibroblasts to fibronectin | | | | | | | | | | | | | |
| Contact time [s] | |  | | 5 | | 20 | | 60 | | 120 | | 240 | | | 360 | | |
| Fibroblasts to FNIII7-10ΔRGD | | *P* value | | 0.010 | | 0.070 | | 0.054 | | 0.792 | | 0.609 | | | 0.475 | | |

Table S4. **Statistical analysis comparing the adhesion force of EDTA- and non-treated HeLa cells or fibroblasts to indicated substrates with given compression force and contact time**. *P* values compare adhesion force of EDTA- and non-treated HeLa cells or fibroblasts to indicated substrate at the given compression force and contact time. *P* values were calculated using two-sided Mann-Whitney tests, black values show significant difference (*P* < 0.05), red values non-significant difference (*P* ≥ 0.05). Data taken from Figure 1C,D and 2A,B.

| **Comparison of the adhesion force of HeLa cells to collagen I patterns with BSA substrates or collagen I patterns under 10 nN compression force** | | | | | | | |
| --- | --- | --- | --- | --- | --- | --- | --- |
| **BSA substrate** |  |  | | | | | |
| Contact time [s] |  | 5 | 20 | 60 | 120 | 240 | 360 |
| Patterned area 42.2 μm^2^ | *P* value | 0.040 | 0.018 | 0.015 | 0.197 | 0.216 | 0.049 |
| Patterned area 27.7 μm^2^ | *P* value | 0.002 | 0.016 | 0.054 | 0.112 | 0.297 | 0.087 |
| Patterned area 2.6 μm^2^ | *P* value | 0.019 | 0.009 | 0.081 | 0.196 | 0.153 | 0.070 |
| **Patterned area 42.2 μm^2^** |  |  | | | | | |
| Contact time [s] | | 5 | 20 | 60 | 120 | 240 | 360 |
| Patterned area 27.7 μm^2^ | *P* value | 0.129 | 0.657 | 0.585 | 0.574 | 0.915 | 0.852 |
| Patterned area 2.6 μm^2^ | *P* value | 0.696 | 0.734 | 0.579 | 0.874 | 0.780 | 0.794 |
| **Patterned area 33.2 μm^2^** |  |  | | | | | |
| Contact time [s] | | 5 | 20 | 60 | 120 | 240 | 360 |
| Patterned area 2.6 μm^2^ | *P* value | 0.206 | 0.857 | 0.963 | 0.499 | 0.785 | 0.981 |

Table S5. **Statistical analysis comparing the adhesion force of HeLa cells to printed collagen I patterns with BSA substrate or indicated collagen I substrate area at given contact time under 10 nN compression force**. *P* values compare adhesion force of HeLa cells to printed collagen patterns of given area with that measured to unrestricted BSA substrate or the indicated collagen I pattern area at the given contact time under 10 nN compression force. *P* values were calculated using two-sided Mann-Whitney tests, black values show significant difference (*P* < 0.05), red values non-significant difference (*P* ≥ 0.05). Data taken from Figure 3A.

| **Comparison of the adhesion force of fibroblasts to fibronectin patterns with FNIII7-10ΔRGD substrates or fibronectin patterns under 10 nN compression force** | | | | | | | | | | | | | | | | | | | |
| --- | --- | --- | --- | --- | --- | --- | --- | --- | --- | --- | --- | --- | --- | --- | --- | --- | --- | --- | --- |
| **FNIII7-10ΔRGD substrate** | |  | |  | | | | | | | | | | | | | | | |
| Contact time [s] | |  | | 5 | | | 20 | | 60 | | | 120 | | | 240 | | 360 | | |
| Patterned area 30.5 μm^2^ | | *P* value | | 0.539 | | | 0.831 | | 0.829 | | | 0.650 | | | 0.383 | | 0.523 | | |
| Patterned area 8.4 μm^2^ | | *P* value | | 0.013 | | | 0.070 | | 0.136 | | | 0.052 | | | 0.279 | | 0.759 | | |
| Patterned area 2.3 μm^2^ | | *P* value | | 0.465 | | | 0.823 | | 0.194 | | | 0.282 | | | 0.049 | | 0.197 | | |
| **Patterned area 30.5 [μm^2^]** |  | | |  | | | | | | | | | | | | | | | |
| Contact time [s] | | | | 5 | | | 20 | | 60 | | | 120 | | | 240 | | 360 | | |
| Patterned area 8.4 μm^2^ | | *P* value | | | 0.221 | | | 0.196 | | 0.212 | | | 0.199 | | 0.053 | 0.179 | | | |
| Patterned area 2.3 μm^2^ | | *P* value | | | 0.948 | | | 0.640 | | 0.160 | | | 0.239 | | 0.400 | 0.405 | | | |
| **Patterned area 8.4 [μm^2^]** |  | | |  | | | | | | | | | | | | | | | |
| Contact time [s] | | | | 5 | | | 20 | | 60 | | | 120 | | | 240 | | 360 | | |
| Patterned area 2.3 μm^2^ | | *P* value | 0.190 | | | 0.093 | | | | | 0.006 | | | 0.011 | 0.003 | | | 0.060 |  |

Table S6. Statistical analysis comparing the adhesion force of fibroblasts to printed fibronectin patterns with **FNIII7-10ΔRGD substrate** or indicated fibronectin substrate area at given contact time under 10 nN compression force. *P* values compare adhesion force of fibroblasts to printed fibronectin patterns of given area with that measured of unrestricted FNIII7-10ΔRGD substrate or the indicated fibronectin pattern area at the given contact time under 10 nN compression force. *P* values were calculated using two-sided Mann-Whitney tests, black values show significant differences (*P* < 0.05), and red values non-significant differences (*P* ≥ 0.05). Data taken from Figure 3C.

| **Comparison of the adhesion force of HeLa cells to collagen I substrates** | | | | | | | |
| --- | --- | --- | --- | --- | --- | --- | --- |
| **Compression force [nN]** |  | **1** |  |  |  |  |  |
| Contact time [s] |  | 5 | 20 | 60 | 120 | 240 | 360 |
| Compression force 2 nN | *P* value | 0.002 | <0.001 | 0.082 | 0.006 | 0.205 | 0.004 |
| Compression force 5 nN | *P* value | 0.001 | <0.001 | 0.001 | <0.001 | 0.003 | 0.001 |
| Compression force 10 nN | *P* value | <0.001 | <0.001 | <0.001 | <0.001 | <0.001 | <0.001 |
| **Compression force [nN]** |  | **2** |  |  |  |  |  |
| Contact time [s] |  | 5 | 20 | 60 | 120 | 240 | 360 |
| Compression force 5 nN | *P* value | 0.514 | 0.437 | 0.101 | 0.128 | 0.205 | 0.786 |
| Compression force 10 nN | *P* value | 0.982 | 0.948 | 0.026 | 0.302 | 0.159 | 0.572 |
| **Compression force [nN]** |  | **5** |  |  |  |  |  |
| Contact time [s] |  | 5 | 20 | 60 | 120 | 240 | 360 |
| Compression force 10 nN | *P* value | 0.507 | 0.537 | 0.982 | 0.802 | 0.846 | 0.423 |

Table S7. **Statistical analysis comparing the adhesion force of glycosidase-cocktail treated HeLa cells to collagen I substrates with indicated compression force at given contact time**. *P* values compare adhesion force of glycosidase-cocktail treated HeLa cells to collagen I substrates at given force with that measured at the indicated compression force and contact time. *P* values were calculated using two-sided Mann-Whitney tests, black values show significant differences (*P* < 0.05), and red values non-significant differences (*P* ≥ 0.05). Data taken from Figure 4A.

| **Comparison of the adhesion force of fibroblasts to fibronectin substrates** | | | | | | | |
| --- | --- | --- | --- | --- | --- | --- | --- |
| **Compression force [nN]** |  | **1** |  |  |  |  |  |
| Contact time [s] |  | 5 | 20 | 60 | 120 | 240 | 360 |
| Compression force 2 nN | *P* value | 0.066 | 0.225 | 0.181 | >0.999 | 0.968 | 0.375 |
| Compression force 5 nN | *P* value | 0.022 | 0.130 | 0.148 | 0.403 | 0.320 | 0.698 |
| Compression force 10 nN | *P* value | <0.001 | 0.007 | <0.001 | 0.001 | 0.009 | 0.001 |
| **Compression force [nN]** |  | **2** |  |  |  |  |  |
| Contact time [s] |  | 5 | 20 | 60 | 120 | 240 | 360 |
| Compression force 5 nN | *P* value | 0.256 | 0.183 | 0.611 | 0.454 | 0.434 | 0.395 |
| Compression force 10 nN | *P* value | 0.017 | 0.020 | <0.001 | 0.06 | 0.033 | 0.001 |
| **Compression force [nN]** |  | **5** |  |  |  |  |  |
| Contact time [s] |  | 5 | 20 | 60 | 120 | 240 | 360 |
| Compression force 10 nN | *P* value | 0.281 | 0.470 | 0.003 | 0.029 | 0.548 | 0.001 |

Table S8. **Statistical analysis comparing the adhesion force of** **glycosidase-cocktail treated** **fibroblasts to fibronectin substrates with indicated compression force at given contact time**. *P* values compare adhesion force of glycosidase-cocktail treated fibroblasts to fibronectin at given force with that measured at the indicated compression force and contact time. *P* values were calculated using two-sided Mann-Whitney tests, black values show significant differences (*P* < 0.05), and red values non-significant differences (*P* ≥ 0.05). Data taken from Figure 4C.

| **Comparison of the adhesion strengthening rate of glycosidase-cocktail treated cells** | | | | |
| --- | --- | --- | --- | --- |
| **Comparison of the adhesion strengthening rate of HeLa cells to unrestricted collagen I** | | | | |
| Compression force | | 2 nN | 5 nN |  |
| 5 nN | *P* value | 0.955 |  |  |
| 10 nN | *P* value | 0.049 | 0.093 |  |
| **Comparison of the adhesion strengthening rate of fibroblasts to unrestricted fibronectin** | | | | |
| Compression force | | 2 nN | 5 nN |  |
| 5 nN | *P* value | 0.745 |  |  |
| 10 nN | *P* value | 0.013 | 0.047 |  |

**Table S9. Statistical analysis of the adhesion strengthening rate of glycosidase-cocktail treated cells to substrates.** Adhesion strengthening rate of glycosidase-cocktail treated HeLa cells or fibroblasts as quantified by the slope of a linear regression fit of the adhesion force to collagen I or fibronectin substrates respectively for all contact times. *P* values compare adhesion strengthening rates of glycosidase-cocktail treated HeLa cells to collagen I and of glycosidase-cocktail treated fibroblasts to fibronectin at given compression force with that measured at indicated compression force. *P* values were calculated using extra sum-of-squares *F* test, black values show significant differences (*P* < 0.05), and red values non-significant differences (*P* ≥ 0.05)*.* Data taken from Figure 4E,F.

References

1. Helenius, J., Heisenberg, C.-P., Gaub, H.E., & Muller, D.J., Single-cell force spectroscopy, 2008, *J. Cell Sci.* 121(11):1785–1791, 10.1242/jcs.030999.
